# Natural variation in *OsMYB305* downregulating cytokinin‐mediated inhibition of leaf senescence contributes to regional adaptation in rice

**DOI:** 10.1111/nph.71335

**Published:** 2026-06-04

**Authors:** Boyeong Kim, Jinah Kim, Yejin Shim, Jinku Kang, Hyeryung Yoon, Sung‐Hwan Cho, Nam‐Chon Paek, Kiyoon Kang

**Affiliations:** ^1^ Department of Agriculture, Forestry and Bioresources, Research Institute of Agriculture and Life Sciences Seoul National University Seoul 08826 Republic of Korea; ^2^ Plant Genomics and Breeding Institute Seoul National University Seoul 08826 Republic of Korea; ^3^ Department of Environmental and Biological Chemistry Chungbuk National University Cheongju 28644 Republic of Korea; ^4^ Division of Life Sciences Incheon National University Incheon 22012 Republic of Korea

**Keywords:** cytokinins, leaf senescence, natural variation, OsMYB305, rice

## Abstract

- Accumulating evidence has identified transcription factors (TFs) as key regulators of the onset and progression of leaf senescence. However, the regulatory role of the rice myeloblastosis (MYB) TF OsMYB305 in this process remains largely unknown.
- CRISPR/Cas9‐mediated *osmyb305* knockout mutants were used to examine senescence phenotypes. Protein–DNA interactions were analyzed using *in vivo* and *in vitro* binding assays and predicted using AlphaFold. Natural variations in *OsMYB305* were investigated using the International Rice Genebank Collection Information System database.
- *osmyb305* mutants exhibited delayed leaf senescence under both natural and dark‐induced senescence conditions. OsMYB305 TF downregulated *adenosine phosphate‐isopentenyltransferase 8* (*OsIPT8*) and upregulated *cytokinin oxidase/dehydrogenase 2* (*OsCKX2*) by directly binding to their promoters, thereby modulating cytokinin homeostasis during leaf senescence. Natural single‐nucleotide polymorphisms (SNPs) in *OsMYB305* were associated with phenotypic variation in leaf senescence among 3000 rice accessions, and a SNP in the *cis*‐element of *OsIPT8* promoter disrupted OsMYB305 binding affinity.
- These results identify OsMYB305 as a key transcriptional activator inducing leaf senescence and suggest that natural allelic variation in *OsMYB305* contributes to regional adaptation in rice cultivation.

## Graphical abstract

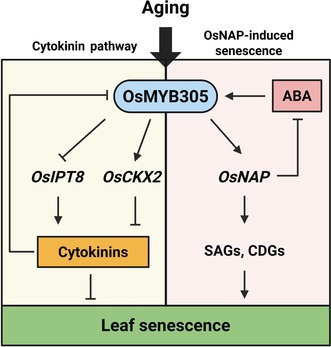

## Introduction

Leaf senescence represents the final stage of leaf development, which is accompanied by Chl degradation and visible leaf yellowing (Kuai *et al*., 2018). This process involves a coordinated series of physiological and biochemical changes, including the dismantling of cellular components, ultimately leading to programmed cell death (Masclaux‐Daubresse *et al*., 2008; Distelfeld *et al*., 2014). In addition, leaf senescence is a highly complex and tightly regulated process, and an equally critical aspect during the reproductive phase is the remobilization of nutrients from source to sink organs, particularly from leaves to developing grains (Thomas & Ougham, 2014; Havé *et al*., 2017). Proper execution of senescence progression has been shown to profoundly influence crop yield and grain quality, underscoring the importance of precisely timed initiation and progression of leaf senescence for optimal agricultural productivity (Uauy *et al*., 2006).

Phytohormones serve as major endogenous regulators of leaf senescence. Senescence progression is promoted by jasmonic acid (JA), ethylene, and abscisic acid (ABA), whereas cytokinins (CKs), gibberellins (GAs), and auxin act to delay this process (Thomas & Ougham, 2014; Ferrante & Carillo, 2025). Among these hormones, ABA has been extensively characterized as a senescence‐promoting signal, in addition to its well‐established functions in abiotic stress tolerance, across diverse plant species, including *Arabidopsis thaliana* and rice (*Oryza sativa*) (Lee *et al*., 2011; Wang *et al*., 2016). Exogenous application of ABA accelerates leaf senescence by inducing the transcription of senescence‐associated genes (SAGs) and Chl degradation genes (CDGs) (Yang *et al*., 2014). ABA is biosynthesized from xanthoxin through the catalytic activity of 9‐*cis*‐epoxycarotenoid dioxygenases (NCEDs), key rate‐limiting enzymes in the ABA biosynthetic pathway. Consistently, overexpression of *OsNCED5* elevates endogenous ABA levels and accelerates leaf senescence in rice (Tan *et al*., 2003; Huang *et al*., 2019).

In contrast to the senescence‐promoting role of ABA, CKs delay leaf senescence by modulating carbohydrate metabolism, promoting Chl synthesis, and sustaining photosynthetic activity (Distelfeld *et al*., 2014; Wu *et al*., 2021). CKs are purine‐derived phytohormones synthesized from adenine and are characterized by either an aromatic side chain or an isoprenoid side chain (Kieber & Schaller, 2018). Among the biologically active forms, N^6^‐(Δ^2^‐isopentenyl) adenine (iP), *trans*‐zeatin (tZ), and dihydrozeatin (DHZ) are major species, generally exhibiting higher abundance and bioactivity. By contrast, the physiological roles of *cis*‐zeatin (cZ) appear to be context‐dependent and remain less fully characterized (Kudo *et al*., 2012; Kieber & Schaller, 2018; Zhao *et al*., 2024). CKs are transported primarily as zeatin ribosides, with tZR and cZR preferentially transported through the xylem and phloem, respectively (Zhao *et al*., 2024). Isoprenoid CKs are synthesized through the catalytic activity of isopentenyl transferases (IPTs) (Zhao *et al*., 2024). Expression of bacterial (*Agrobacterium tumefaciens*) *IPT* driven by the *Arabidopsis* SAG *12* (*SAG12*) promoter increases endogenous CK levels specifically in senescing tobacco (*Nicotiana tabacum*) leaves, resulting in delayed leaf senescence (Gan & Amasino, 1995). In rice, 10 *IPT* genes (*OsIPT1* to *OsIPT10*) have been identified. Eight of these genes (*OsIPT1* to *OsIPT8*) are involved in the biosynthesis of iP‐ and tZ‐type CKs, whereas *OsIPT9* and *OsIPT10* contribute to cZ‐type CK biosynthesis (Gao *et al*., 2019). Conversely, CKs are irreversibly degraded to adenine derivatives by CK oxidase/dehydrogenases (CKXs), the only known enzymes responsible for CK degradation (Takei *et al*., 2001; Werner *et al*., 2003). In rice, RNAi‐mediated downregulation of *Gn1a*/*OsCKX2* delays leaf senescence, whereas *OsCKX11* promotes Chl degradation and mediates antagonistic interactions between CKs and ABA during senescence (Yeh *et al*., 2015; Zhang *et al*., 2021). Although the roles of CKs in delaying leaf senescence are well‐established, the upstream regulatory mechanisms that control CK metabolic genes during senescence remain poorly understood in plants.

The roles of transcription factors (TFs) in regulating leaf senescence have been extensively investigated. In Arabidopsis, *AtMYBR1/AtMYB44* regulates leaf senescence by directly activating SAGs (Jaradat *et al*., 2013). *MYB59* represses *SAG18* during senescence and regulates endogenous phytohormone levels, including salicylic acid (SA) and JA (He *et al*., 2023). In rice, *OsNAP* is upregulated by ABA and directly activates the expression of CDGs and SAGs, such as *STAY‐GREEN* (*OsSGR*), *NONYELLOW COLORING 3* (*OsNYC3*), and *Osl57*, thereby accelerating leaf senescence (Liang *et al*., 2014). *OsMYB102* has been identified as a negative regulator that delays leaf senescence by modulating ABA catabolism and signaling (Piao *et al*., 2019). More recently, *OsMYB305* was reported to enhance nitrogen uptake and assimilation, thereby improving growth under low‐nitrogen conditions (Wang *et al*., 2020). *OsMYB8*, which corresponds to *OsMYB305*, participates in JA biosynthesis and forms the *OsMYB8*‐*Jasmonate Resistant 1* module to regulate diurnal floret opening time in rice (Gou *et al*., 2024a). However, the molecular mechanisms by which *OsMYB305* regulates leaf senescence remain unclear.

In this study, we demonstrated that *OsMYB305* expression increases during both natural senescence (NS) and dark‐induced senescence (DIS) conditions. Genome‐edited *OsMYB305* knockout mutants exhibited delayed leaf senescence under both NS and DIS. *OsMYB305* transcript levels were induced by ABA but repressed by CK treatments. *OsMYB305* mutants exhibited reduced sensitivity to ABA and suppressed ABA signaling during DIS. Moreover, *osmyb305* mutation led to upregulation of CK biosynthetic genes (*OsIPT5* and *OsIPT8*) and downregulation of CK catabolic genes (*OsCKX2*, *OsCKX4*, *OsCKX9*, and *OsCKX11*), resulting in elevated endogenous CK levels in the mutants. Notably, natural variation analysis revealed that among three haplotypes, the *OsMYB305a* allele is preferentially enriched in rice varieties cultivated in high‐latitude regions. Collectively, these findings indicate that *OsMYB305* promotes leaf senescence by coordinating CK homeostasis and ABA signaling, and that its natural allelic variation contributes to regional adaptation in rice.

## Materials and Methods

### Plant materials and rice transformation

The rice of temperate regions (*Oryza sativa* L. *japonica* cv. Dongjin) was used as the wild‐type (WT) parental line. To generate CRISPR/Cas9‐mediated mutants of *OsMYB305* (*osmyb305‐1* and *osmyb305‐2*), one target sequence in the *OsMYB305* coding region was selected for single‐guide RNA (sgRNA) (5′‐GCGGTGCGCAAGGGCCCGTG‐3′) using the CRISPR Direct program (https://crispr.dbcls.jp/) (Naito *et al*., 2015). The target sequence was introduced into the pOs‐sgRNA vector, and the cassette was transferred to the destination vector pH‐UbiCas9‐7 (Miao *et al*., 2013) using Gateway LR Clonase II Enzyme Mix (Invitrogen, Carlsbad, CA, USA). For overexpression lines of *OsMYB305* (*OsMYB305*‐OE1 and *OsMYB305*‐OE2), *OsMYB305* cDNA was amplified using gene‐specific primers listed in Supporting Information Table S1 and subcloned into pCR™8/GW/TOPO to ligate into pMDC32 Gateway binary vectors containing the *35S* promoter. Each vector construct was introduced into the WT (cv. Dongjin) calli derived from mature seed embryos via *Agrobacterium*‐mediated transformation (strain LBA4404) (Lee *et al*., 2006). The transgenic rice plants were selected by hygromycin and confirmed by genomic PCR using specific primers in Table S1. The *osmyb305‐3* T‐DNA insertion line (OsMYB8/OsMYB305; LOC_Os01g45090; PFG_2C‐40 159) mutant, generated in the cv. Dongjin background, was obtained from the Crop Biotech Institute at Kyung Hee University, Republic of Korea (Jeon *et al*., 2000).

### Plant growth conditions

The WT and genome‐edited *osmyb305* mutants were grown in a paddy field under natural long‐day (NLD, > 14‐h light : day) conditions in Suwon, Republic of Korea (37° N latitude, 127° E longitude). For dark or phytohormone treatment to detached leaves, leaves were floated on a 3 mM MES buffer (pH 5.8) with abaxial side up and incubated in complete dark conditions at 28°C or 3 mM MES buffer containing phytohormone under continuous white light conditions (CL; 30°C, light intensity of 100 μmol m^−2^ s^−1^). For phytohormone treatments in rice seedlings, sterilized WT seeds were grown on 1/2 Murashige and Skoog (MS) phytoagar medium for 10 d under long‐day condition (LD; 14‐h light : day, 30°C : 25°C day : night temperature, light intensity of 100 μmol  m^−2^ s^−1^) and then transferred to 1/2 MS liquid medium supplemented with the phytohormones for 1 h under LD condition. To examine ABA sensitivity, seedlings were grown for 3 d on 1/2 MS phytoagar medium under CL condition and subsequently transferred to 1/2 MS medium containing 0, 2.5, or 5‐μM ABA under LD condition.

### Promoter β‐glucuronidase (GUS) assay

The promoter region of *OsMYB305* was ligated into pMDC163 Gateway binary vector, which contains the β‐glucuronidase (GUS) reporter gene. The construct was introduced into calli derived from mature WT (cv. Dongjin) seed embryos using *Agrobacterium* strain LBA4404. GUS assays were performed as described previously (Liu *et al*., 2017). The plant tissues were histochemically stained in GUS chromogenic solution (10 mM EDTA (pH 8), 50 mM phosphate buffer, 0.1% Triton X‐100, 1 mM potassium ferrocyanide, 1 mM potassium ferricyanide, 20% methanol, 2 mM X‐gluc, and sterile distilled water) and incubated at 37°C. Subsequently, the samples were immersed in an ethanol solution to clear Chl and visualized using a stereomicroscope (version ZEISS SteREO Discovery. V12, ZEISS).

### Determination of total Chl content and maximum photosystem (PSII) efficiency

ChI was extracted using 80% acetone, and the extracts were centrifuged at 15 000 *g* for 10 min at 10°C. The absorbance of the supernatants was measured at 647 nm and 663 nm using a spectrophotometer (BioTek Instruments, Winooski, VT, USA). The *F*_v_/*F*_m_ ratio was measured using an OS‐30p + instrument (Opti‐Sciences, Hudson, NH, USA). The middle sections of flag leaves (FLs) grown in a paddy field were used for measurements. Leaves were dark‐adapted for 5 min before determining the *F*_v_/*F*_m_ ratio.

### Reverse transcription quantitative PCR (RT‐qPCR) analysis

Total RNA was extracted with GeneAll Hybrid‐R (GeneAll Biotechnology, Seoul, Republic of Korea). First‐strand cDNA was synthesized with 2 μg of total RNA using M‐MLV reverse transcriptase and oligo(dT)_18_ primers (Macrogen, Seoul, Republic of Korea). For reverse transcription quantitative PCR (RT‐qPCR), a mixture was prepared including first‐strand cDNA mixture, 2 × GoTaq PCR Mix (Promega) and 10 pmol qRT primer pairs (Table S1). The reactions were performed using a LightCycler 2.0 instrument (Roche). *OsUBQ5* was used as an internal control and the relative expression level was calculated using the 2^−ΔΔCT^ method as previously described (Livak & Schmittgen, 2001).

### Determination of endogenous phytohormone contents

Leaf samples at 30 d after heading (DAH) were used for phytohormone quantification. The endogenous CK levels were extracted as previously described (Zhu *et al*., 2020). Briefly, 100 mg of homogenized and lyophilized FL tissues were mixed with 10 ml extraction buffer (methanol/water/formic acid (15/4/1, v/v/v)) and centrifuged 10 000 g for 20 min. The supernatant was loaded onto Sep‐Pak C18 Cartridge System (Waters Associates Inc., Milford, MA, USA), washed with washing buffer (methanol/water/formic acid (10/95/0.1, v/v/v)), and subsequently eluted with elution buffer (methanol/water/formic acid (85/15/0.1, v/v/v)). To quantify endogenous ABA content, 50 mg of FLs was sampled according to previously described methods with minor modifications (Salem *et al*., 2020). Leaves were ground with 0.8 ml of 0.1% HCl and shaken for 30 min at 4°C, followed by 15‐min sonication. Subsequently, 1 ml of methyl‐tert‐butyl‐ether was added and shaken for 30 min at 4°C. After centrifuging 10 000 ***g*** for 10 min at 4°C, the supernatant was dried by Speed Vac (Hanil Science Industrial, Daejeon, Republic of Korea) for 1 h. The pellet was resuspended in 100 μl of solution (water/methanol (1/1, v/v)). To quantify CKs and ABA, each extract was analyzed using UHPLC (Ultimate 3000, Dionex, Sunnyvale, CA, USA) with Hypersil GOLD 1 Column (50 × 4.6 mm, 3 μm) (Thermo Fisher Scientific, Cheshire, UK).

### Determination of CKX activity

The CKX activity was determined based on colorimetric method (Joshi *et al*., 2018) and leaf samples were collected at 30 DAH. Briefly, 500 mg FL tissue was ground and mixed with 3 ml buffer (50 mM potassium acetate, 1 mM MgSO_4_·7H_2_O, 2 mM CaCl_2_·2H_2_O, 0.5 mM dithiothreitol, and a protease inhibitor cocktail (Roche)). Following centrifugation (18 000 *g* for 30 min at 4°C), 0.015% of an aqueous solution (5% polyethyleneimine, 0.5 mM phenylmethanesulfonyl fluoride, and 0.5 mM Nα‐tosyl‐L‐lysine chloromethyl ketone) was added to 1 ml of supernatant. The solution was centrifuged under the same conditions, and the resulting pellet was discarded. A 200 μl aliquot of the supernatant was added to 400 μl of reaction buffer (75 mM Tris–HCl (pH 8.5), 0.5 mM dichlorophenolindophenol, and 0.15 mM iP substrate). After incubation at 37°C for 1 h, the reaction was terminated by adding 300 μl of 40% trichloroacetic acid (TCA). The mixture was then centrifuged at 15 000 *g* for 5 min at 4°C. Subsequently, 200 μl of 2% 4‐aminophenol, prepared in 6% TCA, was added to the supernatant, and the mixture was incubated at room temperature for 10 min. To detect Schiff base formation, the absorbance was measured at 352 nm using a UV/VIS spectrophotometer (BioTek Instruments) and specific CKX activity was expressed as pkat mg^−1^ protein. The protein content of the extracts was determined using Bradford reagent (Sigma‐Aldrich).

### Y1H and transcriptional activity assays

The full‐length coding sequence (CDS) of *OsMYB305* was cloned into pGADT7, pGBKT7, or rGAL4 vectors (Ohta *et al*., 2001). Each region of possible target genes was amplified and inserted into the pLacZi vector. The plasmids were transformed into yeast (*Saccharomyces cerevisiae*) strains YM4271 and AH109 for yeast one‐hybrid (Y1H) and transcriptional activity assays, respectively, using the polyethylene/lithium acetate (PEG/LiAc) method. Subsequently, GUS liquid assays were performed according to the Yeast Protocols Handbook (Clontech, Mountain View, CA, USA) using chlorophenol red‐β‐D‐galactopyranoside (CPRG, Roche) as substrate. The absorbance of extracts was measured at a wavelength of 574 nm using a UV/VIS spectrophotometer (BioTek Instruments).

### ChIP‐qPCR assay

The *pUbi*::GFP and *pUbi*::OsMYB305‐GFP plasmids were cloned for ChIP‐qPCR assay and each construct was transfected into rice protoplasts as described previously (Zhang *et al*., 2011). After 16‐hr incubation in dark conditions, protoplasts were cross‐linked with 1% formaldehyde for 20 min under vacuum. After nuclei isolation and lysis, the chromatin complexes were purified and fragmented by sonication using a VCX500 ultrasonic processor (Sonics, Newtown, USA). Anti‐GFP antibodies (Abcam, Cambridge, UK) and protein A agarose beads (Millipore, Billerica, MA, USA) were used to isolate the protein–chromatin complexes. The DNA was recovered and purified using a Gel and PCR Clean‐Up Kit (Cosmogenetech, Seoul, Republic of Korea). RT‐qPCR analysis was conducted in a LightCycler 2.0 instrument (Roche). *Actin* was used as a negative control and gene‐specific primers are listed in Table S1.

### Electrophoretic mobility shift assays (EMSA)

To purify the OsMYB305‐His protein (containing six consecutive histidine residues), the CDS of *OsMYB305* was cloned into the pET21a vector and expressed in *Escherichia coli* Rosetta 2 (DE3) cells, then induced with 0.5 mM isopropyl β‐D‐thiogalactoside at 16°C for 16 h. Cells were harvested and lysed in buffer (50 mM sodium phosphate (pH 7.4), 0.5 M NaCl, 0.2 M PMSF, 1 mg ml^−1^ lysozyme, 10% NP‐40, and a protease inhibitor cocktail). The recombinant protein was purified using ProBond resin (Novex, San Diego, CA, USA) and a Poly‐Prep chromatography column (Bio‐Rad). For 5′‐biotin‐labeled or unlabeled probes, primers listed in Table S1 were annealed. Electrophoretic mobility shift assay (EMSA) was performed using the LightShift Chemiluminescent EMSA Kit (Thermo Fisher Scientific) and detected with the Chemiluminescent Nucleic Acid Detection Module (Thermo Fisher Scientific).

### Dual‐luciferase (LUC) assays in rice protoplasts

The promoter regions of the target genes were cloned into a pJD301 vector for generating plasmid containing the LUC reporter gene using In‐Fusion HD Cloning Kit (Takara, Otsu, Shiga, Japan). As the effector plasmid, the full length of the CDS of *OsMYB305* was inserted into pGA3651 vector. Renilla luciferase was used as an internal control. Each combination of reporter, effector, and internal control plasmids was cotransfected into rice protoplasts using PEG‐mediated transfection (Zhang *et al*., 2011). After incubation in the dark at 25°C for 16 hr, the LUC activity of each cell lysate was measured using a dual‐luciferase (LUS) reporter assay system kit (E1910, Promega).

### Single‐nucleotide polymorphisms (SNPs) analysis

The SNPs in the *OsMYB305* CDS and the promoters of *OsIPT8*, *OsCKX*2, and *OsNAP* were investigated using public databases. For cultivated rice (*O. sativa*), SNP data were retrieved from the 3 K Rice Genome Project via SNP‐Seek platform (IRGCIS; Mansueto *et al*., 2017). For wild rice (*Oryza rufipogon*), SNP data were obtained from OryzaGenome Release 2.0 (National Institute of Genetics, Japan; Huang *et al*., 2012). The japonica ‘Nipponbare’ allele served as the reference genome for all analyses. The leaf senescence phenotypic values were also obtained from the IRGCIS. The violin plots were generated using the ggplot2 package in R software (v.4.4.3) to compare the distribution of leaf senescence values across the haplotype.

### Structural and bioinformatics analyses

Structural modeling of *in silico* protein–DNA complexes between OsMYB305 and *pOsIPT8* haplotypes was performed using the AlphaFold 3 server (https://alphafoldserver.com; Abramson *et al*., 2024). Predicted models generated by AlphaFold 3 were ranked based on the predicted local distance difference test (pLDDT) score and predicted aligned error (PAE). Structural models were visualized using UCSF ChimeraX (v.1.11.1) (Pettersen *et al*., 2021). To predict protein post‐translational modification (PTM) sites, including phosphorylation and SUMOylation, bioinformatic analyses were performed using the NetPhos 3.1 Server (https://services.healthtech.dtu.dk/services/NetPhos‐3.1/) for the identification of putative phosphorylation sites and GPS‐SUMO 2.0 (http://sumosp.biocuckoo.org/) for the prediction of SUMOylation sites and SUMO‐interaction motifs (Blom *et al*., 1999; Gou *et al*., 2024b).

### Statistical analysis

All statistical analyses were conducted using one‐way ANOVA followed by Tukey's honestly significant difference (HSD) *post hoc* test for multiple comparisons (> 3 groups), or Student's *t*‐test for pairwise comparisons (2 groups), as appropriate. Analyses were performed using RStudio (R v.4.4.3; https://www.rstudio.com/). Significant differences were indicated by different letters for Tukey's HSD test (*P* < 0.05) or by asterisks (*, *P* < 0.05; **, *P* < 0.01; ***, *P* < 0.001).

## Results

### Expression patterns of *OsMYB305*

**Fig. 1:**
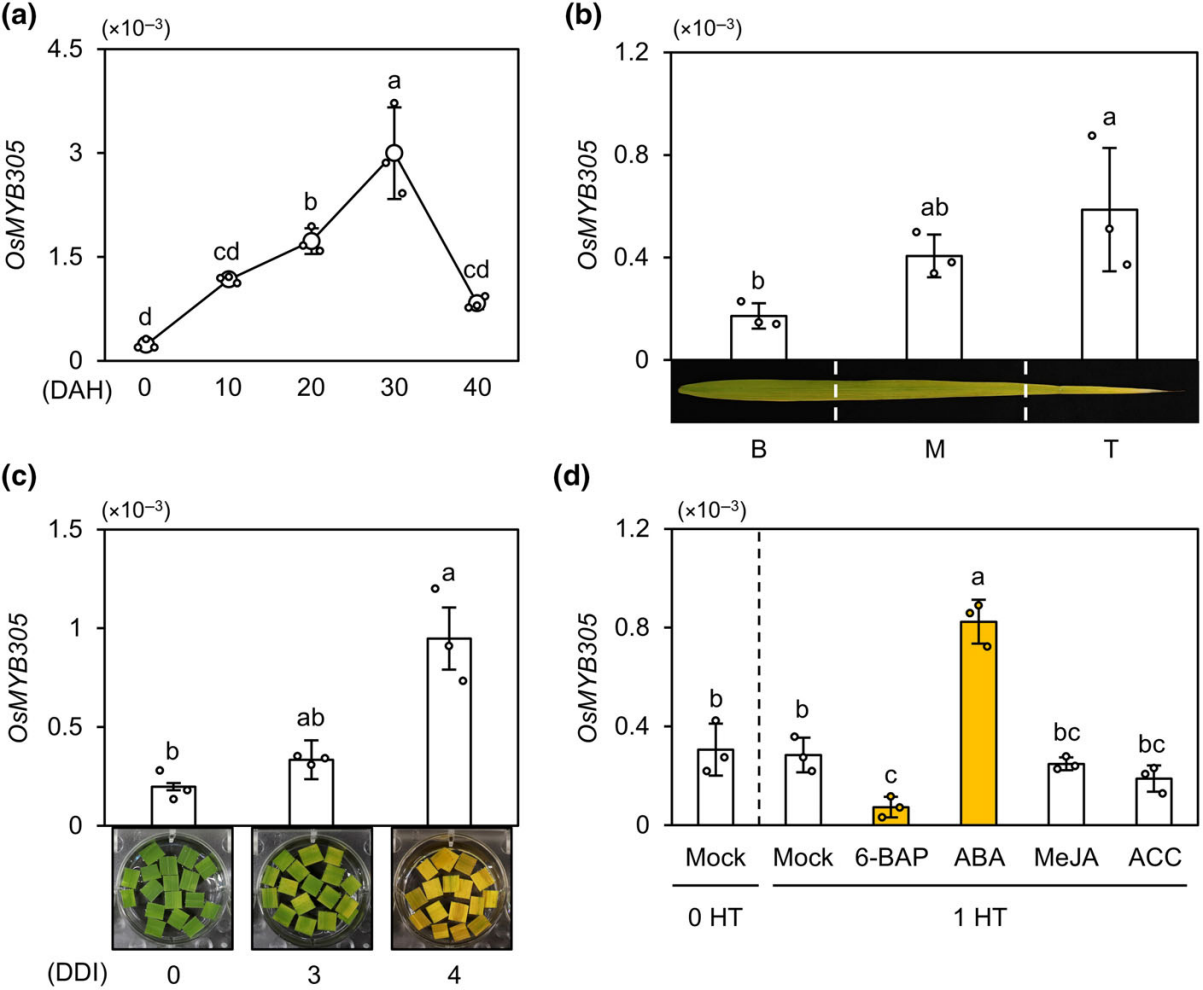
Expression pattern of *OsMYB305*. (a–c) Wild‐type (WT) plants were grown in a paddy field under natural long‐day (LD) conditions in Suwon, Republic of Korea. (a) Flag leaves (FLs) of rice (*Oryza sativa* L.) were sampled at 0–40 d after heading (DAH). (b) FLs of 30 DAH plants were divided into three regions: base (B), middle (M), and tip (T). (c) FLs of 0 DAH were subjected to 0, 3, and 4 d of dark incubation. (d) Ten‐day‐old rice WT seedlings grown on 1/2 Murashige and Skoog (MS) solid medium in a growth chamber under LD conditions were treated with 100 μM abscisic acid (ABA), 100 μM 6‐benzylaminopurine (BAP), 50 μM methyl jasmonic acid (MeJA), or 1 mM 1‐aminocyclopropane‐1‐carboxylic acid (ACC) for 1 h. WT seedlings grown on 1/2 MS medium without hormone supplementation were used as mock controls. Relative transcript levels of *OsMYB305* were determined by reverse transcription‐quantitative PCR (RT‐qPCR) and normalized to rice *UBIQUITIN5* (*OsUBQ5*) using the 2^−ΔΔCT^ method. Data are presented as means ± SD (*n* = 3 biological replicates), with each replicate consisting of four plants per genotype. Statistical analyses were performed using one‐way ANOVA followed by Tukey's honestly significant difference test, with different letters indicating significant differences among groups within the same treatment (*P* < 0.05). HT, hours after treatment.

To investigate *OsMYB305* expression in response to leaf senescence, WT plants were grown in a paddy field in Suwon, Republic of Korea, under NLD conditions. RT‐qPCR analysis revealed that *OsMYB305* transcript levels gradually increased in the FLs until 30 DAH and subsequently declined during NS (Fig. 1a). FLs detached at 30 DAH were divided into base (B), middle (M), and tip (T) regions, which exhibited spatially distinct senescence progression. RT‐qPCR analysis showed that *OsMYB305* transcript levels were higher in the yellowing tip regions than in the green basal regions (Fig. 1b). We further examined *OsMYB305* expression during DIS. FLs detached at 0 DAH were incubated in 3 mM MES buffer (pH 5.8) under complete darkness at 30°C. *OsMYB305* expression was substantially increased up to 4 d of dark incubation (DDI) (Fig. 1c). Next, to examine the expression patterns of *OsMYB305* in response to senescence‐associated phytohormones, 10‐d‐old WT seedlings grown on 1/2 MS medium were treated for 1 h with 100 μM 6‐benzylaminopurine (6‐BAP, a synthetic CK), 100 μM ABA, 50 μM methyl jasmonate (MeJA), or 1 mM 1‐aminocyclopropane‐1‐carboxylic acid (ACC, a precursor of ethylene). RT‐qPCR analysis showed that *OsMYB305* transcript levels were significantly downregulated by 6‐BAP but upregulated by ABA (Fig. 1d). There were no significant differences in *OsMYB305* expression by the treatments of MeJA or ACC. These results indicate that *OsMYB305* is involved in leaf senescence regulation associated with CK and ABA signaling pathways.

To further investigate the spatial expression pattern of *OsMYB305*, WT plants were grown in a growth chamber under LD conditions for 10 d after germination and in a paddy field under NLD conditions for 120 DAS. Tissues were collected from leaf (L), leaf sheath (LS), and root (R) at the vegetative stage, and from FL, leaf blade (LB), LS, panicle (P), node (N), internode (IN), tiller base (TB), and R at the reproductive stage. RT‐qPCR analysis showed that *OsMYB305* expression was generally higher during the reproductive stage, with particularly strong expression in the LS, P, TB, and R tissues (Fig. S1a). Consistent with these findings, GUS staining using transgenic plants carrying the *OsMYB305* promoter region (−2150 to −1‐bp upstream from the start codon ATG) fused to GUS revealed strong GUS activity in P and LS tissues at the reproductive stage, especially in vascular tissues surrounding the phloem, including companion cells (Fig. S1b).

### *osmyb305* mutants exhibited delayed leaf senescence during both natural and dark‐induced senescence

To investigate the roles of *OsMYB305* in leaf senescence, we generated two independent CRISPR/Cas9‐mediated knockout mutants (*osmyb305‐1* and *osmyb305‐2*) and two *OsMYB305*‐overexpressing lines (*OsMYB305*‐OE1 and O*sMYB305*‐OE2). In addition, a T‐DNA insertion mutant (*osmyb305‐3*) was obtained from the RiceGE database (https://signal.salk.edu/cgi‐bin/RiceGE) (Fig. S2a). CRISPR/Cas9‐mediated mutagenesis resulted in a 28‐bp deletion followed by a C‐to‐G substitution and a 1‐bp A insertion in the first exon of *OsMYB305*, causing frameshift mutations in *osmyb305‐1* and *osmyb305‐2*, respectively (Fig. S2b). Semi‐quantitative RT‐PCR analysis revealed that *OsMYB305* transcripts were abundant in the FLs of WT but undetectable in those of *osmyb305‐3* at 0 DAH, indicating that *osmyb305‐3* represents a null mutant (Fig. S2c). Consistently, RT‐qPCR analysis confirmed that *OsMYB305* transcript levels were significantly elevated in the FLs of *OsMYB305*‐OE plants at 0 DAH compared with those of WT (Fig. S2d).

**Fig. 2:**
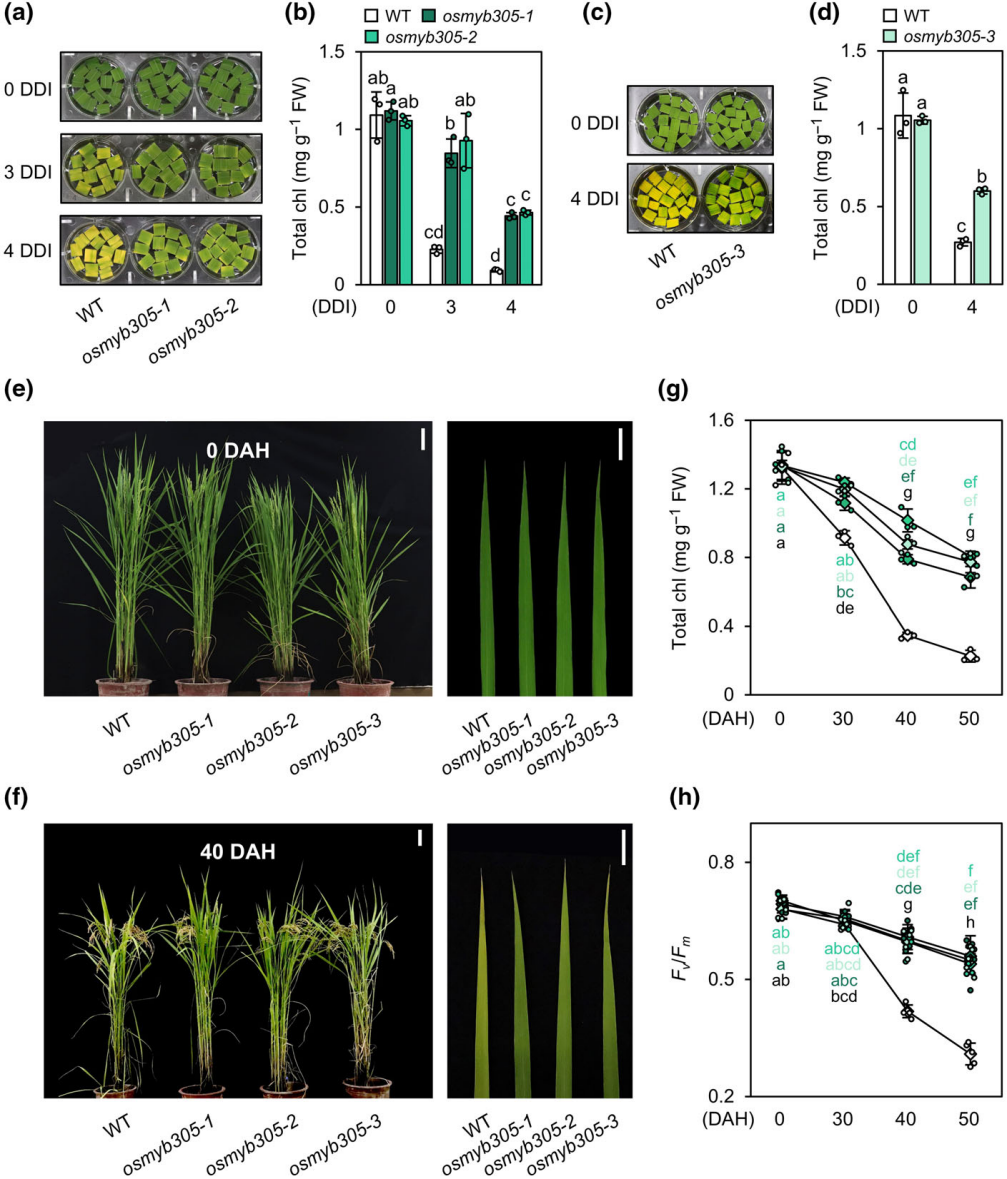
Loss of *OsMYB305* delays natural and dark‐induced senescence. (a–d) Detached flag leaves (FLs) of rice wild‐type (WT) and *osmyb305* mutants (*osmyb305‐1*, *osmyb305‐2*, and *osmyb305‐3*) were subjected to 0, 3, and 4 d of dark incubation. Representative phenotypes (a, c) and total Chl contents (b, d). These experiments were independently repeated three times using separate sets of plates, with similar results. (e–h) Rice plants were grown in a paddy field under natural long‐day conditions. Natural senescence phenotypes (e, f), total Chl contents (g), and maximum quantum efficiency of photosystem II (*F*_v_/*F*_m_) (h). (e, f) Photographs of whole plants and flag leaves were taken at 0 and 40 d after heading (DAH). Bars, 10 cm (whole plants) and 3 cm (FLs). Data are presented as mean ± SD (*n* = 3 biological replicates, each consisting of four plants per genotype (b, d, g), *n* = 6 independent plants per genotype (h)). Statistical analyses were performed using one‐way ANOVA followed by Tukey's honestly significant difference test, with different letters indicating significant differences among groups within the same treatment (*P* < 0.05). FW, fresh weight.

We next examined DIS phenotypes using the FLs detached from WT, *osmyb305* mutants, and *OsMYB305*‐OE plants at 0 DAH. Compared with WT, *osmyb305* mutants exhibited delayed leaf yellowing at 3 and 4 DDI (Fig. 2a,c), whereas *OsMYB305*‐OE plants displayed accelerated leaf yellowing at 3 DDI (Fig. S3a). We further analyzed their senescence phenotypes under NS conditions in a paddy field. The flowering time of WT was comparable to that of *osmyb305* mutants (Fig. S4). No obvious phenotypic differences were observed between WT and *osmyb305* mutants at 0 DAH. However, at 40 DAH, *osmyb305* mutants exhibited delayed senescence compared with WT (Fig. 2e,f). Consistent with these visual observations, total Chl contents were significantly higher in *osmyb305* mutants than in WT during both DIS and NS (Fig. 2b,d,g), whereas *OsMYB305*‐OE plants showed reduced total Chl contents compared with WT at 3 DDI (Fig. S3b). Moreover, although WT plants exhibited a pronounced decline in the *F*_v_/*F*_m_ ratio (an indicator of maximum photosystem II (PSII) efficiency) during NS, *osmyb305* mutants maintained significantly higher *F*_v_/*F*_m_ values (Fig. 2h). Collectively, loss of *OsMYB305* function delays leaf senescence by enhancing Chl retention and maintaining photosynthetic efficiency.

To elucidate the molecular mechanisms by which *OsMYB305* regulates leaf senescence, we examined the expression of *OsNAP* and *OsNAP*‐regulated CDGs and SAGs, including *OsSGR*, *OsNYC3*, and *OsI57*, during DIS and NS. FLs detached from WT and *osmyb305* mutants were subjected to DIS by incubation in 3 mM MES buffer (pH 5.8) under complete darkness at 30°C. In parallel, gene expression was analyzed in their FLs collected during NS. RT‐qPCR analysis revealed that transcript levels of these genes were markedly induced in WT plants during DIS at 4 DDI and NS at 40 DAH compared to 0 DDI and 0 DAH, respectively. In addition, the expression of these genes was significantly reduced in *osmyb305* mutants compared with WT at both 4 DDI and 40 DAH (Fig. S5). These findings indicate that *OsMYB305* functions as a positive regulator of leaf senescence in rice.

We further evaluated agronomic traits in WT and *osmyb305* mutants cultivated in a paddy field in Suwon, Republic of Korea, under NLD conditions. Both plant height and panicle length were significantly reduced in *osmyb305* mutants (Fig. S6a,b). By contrast, no significant differences were observed in the number of panicles per plant or 500‐grain weight between WT and *osmyb305* mutants (Fig. S6c,d). However, loss of *OsMYB305* function resulted in fewer grains per panicle and reduced spikelet fertility (Fig. S6e,f), ultimately leading to a significant reduction in grain yield per plant in *osmyb305* mutants relative to WT (Fig. S6g).

### Loss of *OsMYB305* enhances CK accumulation and reduces ABA sensitivity

**Fig. 3:**
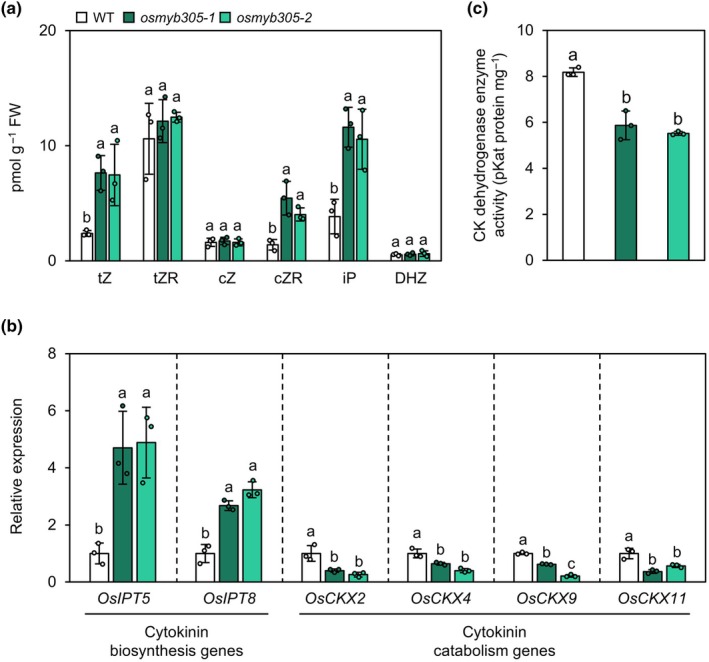
Increased cytokinin levels in *osmyb305* mutants. (a–c) Rice wild‐type (WT) and *osmyb305* mutants were grown in a paddy field under natural long‐day conditions. Flag leaves collected at 30 d after heading were used to quantify endogenous cytokinin (CK) levels (a), analyze the expression of CK biosynthetic (*OsIPT5* and *OsIPT8*) and catabolic (*OsCKX2*, *OsCKX4*, *OsCKX9*, and *OsCKX11*) genes (b), and measure CK dehydrogenase activity (c). Relative transcript levels of CK biosynthetic and catabolic genes were determined by reverse transcription quantitative PCR (RT‐qPCR) and normalized to rice *UBIQUITIN5* (*OsUBQ5*) using the 2^−ΔΔCT^ method. Data are presented as mean ± SD (*n* = 3 biological replicates), with each replicate consisting of eight (a, c) or four (b) plants per genotype. Statistical analyses were performed using one‐way ANOVA followed by Tukey's honestly significant difference test, with different letters indicating significant differences among groups within the same treatment (*P* < 0.05). cZ, *cis*‐zeatin; cZR, *cis*‐zeatin riboside; DHZ, dihydrozeatin; FW, fresh weight; iP, isopentenyladenine; tZ, *trans*‐zeatin; tZR, *trans*‐zeatin riboside.

To assess the role of *OsMYB305* in phytohormone biosynthesis during NS, endogenous CK levels were quantified in FLs of WT and *osmyb305* mutants at 30 DAH using ultra high‐performance liquid chromatography (UHPLC). Quantitative analysis revealed that, among CKs, tZ, cZR, and iP accumulated to significantly higher levels in *osmyb305* mutants than in WT. By contrast, no significant differences were detected in tZR, cZ, and DHZ between WT and *osmyb305* mutants (Fig. 3a). We next examined the expression of genes involved in CK biosynthesis and catabolism. Transcript levels of the CK biosynthetic genes *OsIPT5* and *OsIPT8* were significantly elevated in *osmyb305* mutants compared with WT at 30 DAH. Conversely, expression of CK catabolic genes *OsCKX2*, *OsCKX4*, *OsCKX9*, and *OsCKX11* was markedly reduced in *osmyb305* mutants (Fig. 3b). In addition, CK dehydrogenase activity assays were performed using crude enzyme extracts from the FLs of WT and *osmyb305* mutants at 30 DAH. Consistent with these transcriptional changes, CKX activity was significantly lower in *osmyb305* mutants than in WT (Fig. 3c). Furthermore, endogenous ABA levels were quantified in WT and *osmyb305* mutants. FLs of *osmyb305* mutants accumulated significantly higher ABA levels than those of WT at 30 DAH (Fig. S7a). Consistent with the increased ABA levels, transcript levels of ABA biosynthetic genes *OsNCED1*, *OsNCED2*, and *OsNCED3* were elevated in FLs of *osmyb305* mutants at 30 DAH. By contrast, no significant differences were observed in the expression of ABA catabolic genes *OsABA8ox1*, *OsABA8ox2*, and *OsABA8ox3* between WT and *osmyb305* mutants (Fig. S7b). Collectively, these results indicate that elevated endogenous CK levels in *osmyb305* mutants contribute to delayed leaf senescence during NS.

To examine the involvement of *OsMYB305* in ABA sensitivity, we assessed seedling growth inhibition in response to exogenous ABA treatment. Three‐day‐old WT and *osmyb305* mutant seedlings were grown on 1/2 MS phytoagar medium supplemented with 2.5 or 5 μM ABA for 7 d. Rice seedlings grown on ABA‐free medium served as the mock control (Fig. S8a). Root length in WT seedlings decreased progressively with increasing ABA concentrations, whereas *osmyb305* mutants exhibited significantly longer roots than WT under ABA treatment (Fig. S8b). These results indicate that loss of *OsMYB305* function confers reduced sensitivity to ABA. We next examined the ABA‐induced leaf senescence in WT and *osmyb305* mutants. Detached FLs of WT and *osmyb305* mutants were incubated in a 3 mM MES buffer (pH 5.8) supplemented with 100 μM ABA under continuous light at 30°C, while leaves incubated in a MES buffer without ABA served as the mock control. At 2 d after treatment (DT), *osmyb305* mutants retained leaf greenness and maintained higher total Chl contents than WT leaves (Fig. S8c,d). To determine whether *OsMYB305* regulates ABA signaling during DIS, we examined the expression of ABA signaling genes, rice *ABA‐responsive element binding factor 2* (*OsABF2*), *OsABF4*, and rice *ABSCISIC ACID INSENSITIVE 5* (*OsABI5*) in the detached leaves of WT and *osmyb305* mutants, as shown in Fig. 2a. RT‐qPCR analysis revealed that transcript levels of these genes were upregulated in WT during DIS, whereas their expression was reduced in *osmyb305* mutants compared with WT at 4 DDI (Fig. S8e–g). Taken together, these results indicate that loss of *OsMYB305* delays leaf senescence by suppressing ABA signaling gene expression.

### OsMYB305 directly represses *OsIPT8* and activates *OsCKX2* and *OsNAP*

**Fig. 4:**
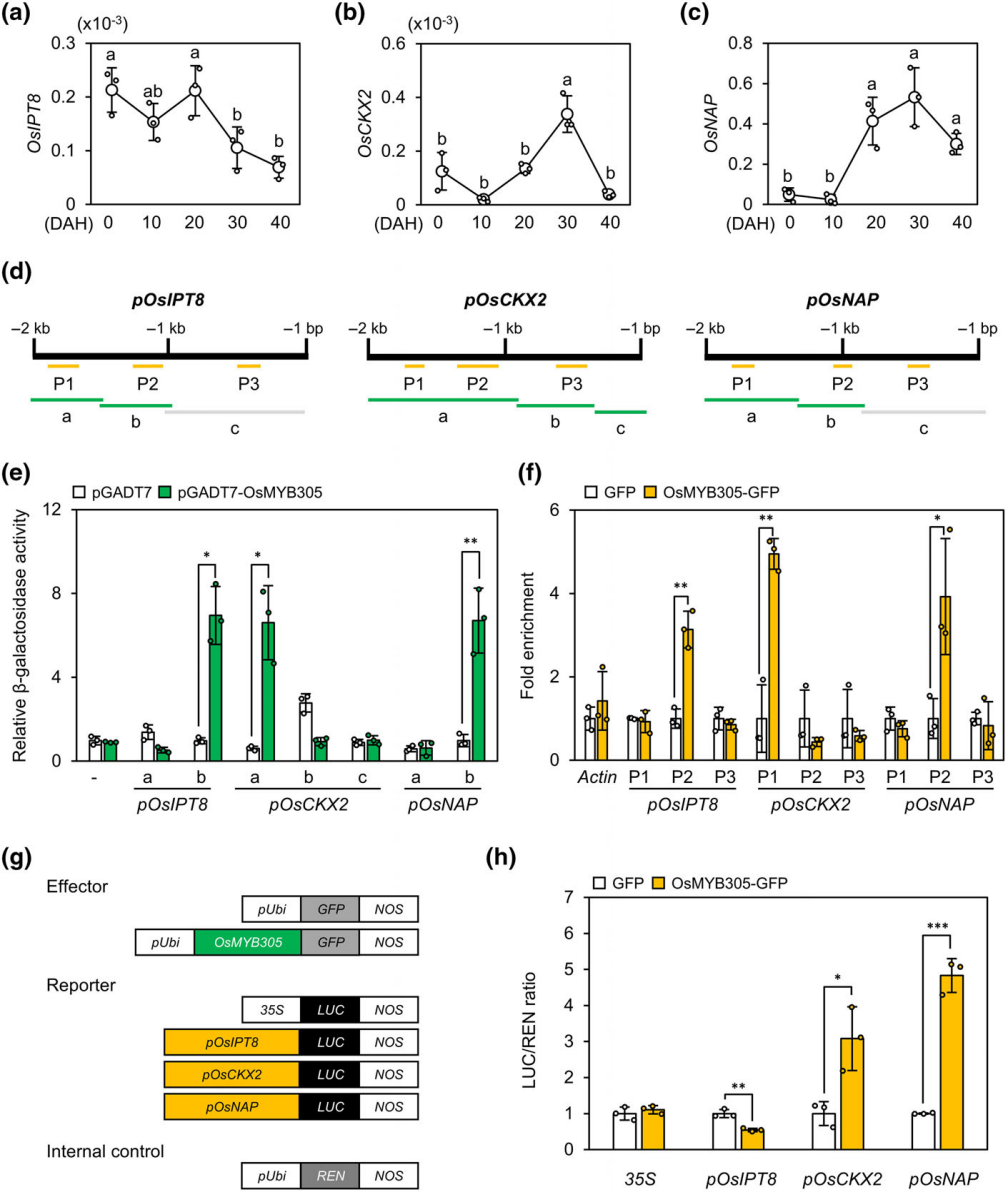
OsMYB305 directly binds to the promoters of *OsIPT8*, *OsCKX2*, and *OsNAP*. (a–c) Rice wild‐type (WT) plants were grown in a paddy field under natural long‐day conditions. Flag leaves were sampled at 0–40 d after heading (DAH). Relative transcript levels of *OsIPT8* (a), *OsCKX2* (b), and *OsNAP* (c) were determined by reverse transcription quantitative PCR (RT‐qPCR) and normalized to rice *UBIQUITIN5* (*OsUBQ5*) using the 2^−ΔΔCT^ method. (d) Schematic representation of the *OsIPT8*, *OsCKX2*, and *OsNAP* promoter regions used for yeast one‐hybrid (Y1H) and chromatin immunoprecipitation (ChIP)‐qPCR assays. Green and yellow horizontal bars indicate promoter fragments used for Y1H and ChIP‐qPCR analyses, respectively. Gray horizontal bars represent fragments that showed self‐activation in the Y1H assay. (e) Y1H assays were determined by measuring β‐galactosidase ( activity using chlorophenol red‐β‐D‐galactopyranoside as a substrate. Empty bait (pLacZi) and prey (pGADT7) vectors (−) were used as negative controls. Relative β‐galactosidase activity was normalized to the negative control. (f) Fold enrichment of ChIP‐qPCR assays was determined following immunoprecipitation with an anti‐GFP antibody. *Actin* was used as a negative control locus. (g) Schematic diagrams of the effector, reporter, and internal control constructs used in the transient dual‐luciferase (LUC) assay. *pUbi*, *Ubiquitin* promoter; *35S*, *35S* promoter; promoter regions of *pOsIPT8*, *pOsCKX2* or *pOsNAP*; LUC, firefly luciferase; REN, Renilla luciferase; NOS, nopaline synthase terminator. (h) LUC activities were calculated as relative firefly LUC to REN ratios. The *35S* promoter‐driven construct was used as a negative control. Data are presented as mean ± SD (*n* = 3 biological replicates, each consisting of four plants per genotype (a–c), and n = 3 independent biological replicates (e, f, h)). Statistical analyses were performed using one‐way ANOVA followed by Tukey's honestly significant difference test for panels (a–c) or Student's *t*‐test (e, f, h). Different letters indicate significant differences among groups within the same treatment (*P* < 0.05), whereas asterisks indicate significant differences (*, *P* < 0.05; **, *P* < 0.01; ***, *P* < 0.001).

We evaluated the expression profiles of *OsIPT8*, *OsCKX2*, and *OsNAP* in the WT FLs during NS, as shown in Fig. 1a. The transcript levels of *OsIPT8* gradually decreased during NS, showing an expression pattern opposite to that of *OsMYB305* (Fig. 4a). By contrast, the expression patterns of *OsCKX2* and *OsNAP* were positively correlated with *OsMYB305* during NS (Fig. 4b,c). To determine whether OsMYB305 directly binds to the promoters of downstream target genes, we performed Y1H and chromatin immunoprecipitation (ChIP)‐qPCR assays. Promoter regions (−2‐kb upstream from the ATG start codon) for each gene were divided into multiple fragments (Figs 4d, S9a). Y1H assays revealed that OsMYB305 directly binds to the promoter fragments of *OsIPT8*, *OsCKX2*, and *OsNAP* (*pOsIPT8*‐b, *pOsCKX2*‐a, and *pOsNAP*‐b) (Fig. 4e). By contrast, no binding was detected to promoter regions of *OsIPT5*, *OsCKX4*, *OsCKX9*, or *OsCKX11* (Fig. S9b). These interactions were further validated *in planta* by ChIP‐qPCR assays. OsMYB305‐GFP showed strong enrichment at the P2 amplicon of *pOsIPT8* (*pOsIPT*‐P2), the P1 amplicon of *pOsCKX2* (*pOsCKX2*‐P1), and the P2 amplicon of *pOsNAP* (*pOsNAP*‐P2) compared with the GFP control (Fig. 4f).

To assess the transcriptional regulatory activity of OsMYB305, we conducted dual‐LUC reporter assays using rice protoplasts. Promoter regions of *pOsIPT8* (−1967 bp upstream from the start codon), *pOCKX2* (−1892 bp), *pOsNAP* (−1873 bp) were fused to the LUC reporter gene (Fig. 4g). Compared with cotransfection with *pUbi*::GFP, LUC activity in rice protoplasts harboring the *pOsIPT8*::*LUC* plasmid was reduced upon cotransfection with *pUbi*::OsMYB305‐GFP, whereas LUC activity was significantly increased in rice protoplasts harboring *pOsCKX2*::*LUC* or *pOsNAP*::*LUC* (Fig. 4h). Thus, OsMYB305 functions as the transcriptional repressor downregulating *OsIPT8* as well as an activator upregulating *OsCKX2* and *OsNAP* (Fig. S10). The dual functionality of OsMYB305 suggests the presence of a molecular switch. To investigate whether PTMs might contribute to this functional versatility, we performed *in silico* analyses of OsMYB305 protein. Putative phosphorylation sites were predicted using NetPhos 3.1 Server (Blom *et al*., 1999). Analysis of serine, threonine, and tyrosine residues identified several potential phosphorylation sites, particularly in the C‐terminal region of OsMYB305, 150 amino acid positions (Fig. S11a). We also examined potential small ubiquitin‐like modifier (SUMO) conjugation (SUMOylation) sites using GPS‐SUMO 2.0 Server (Gou *et al*., 2024b). Two residues were predicted as candidate SUMOylation sites, along with potential SUMO‐interaction motifs (Fig. S11b). These bioinformatic analyses suggest that PTMs may regulate the dual transcriptional activities of OsMYB305. Through this dual regulatory activity, OsMYB305 reduces endogenous CK levels and promotes leaf senescence.

**Fig. 5:**
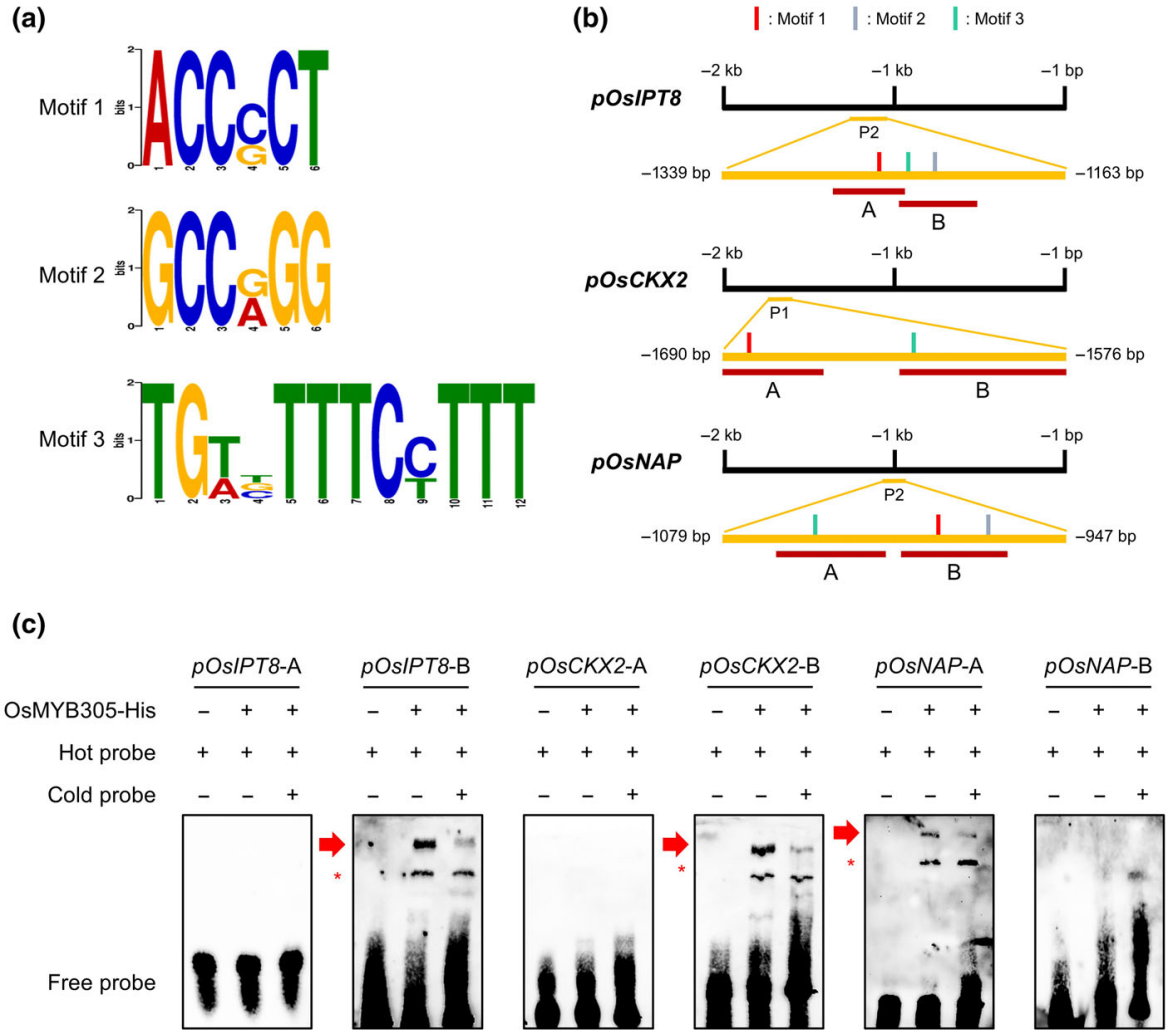
Noncanonical *cis*‐element recognized by OsMYB305. (a) Candidate *cis*‐elements identified by Multiple Em for Motif Elicitation analysis. (b) Schematic representation of the *OsIPT8*, *OsCKX2*, and *OsNAP* promoter regions used for electrophoretic mobility shift assay (EMSA). Yellow horizontal bars represent promoter fragments bound by OsMYB305 protein, as identified by chromatin immunoprecipitation‐quantitative PCR analysis. Red horizontal bars depict fragments used as probes in EMSA. (c) EMSA was conducted using purified OsMYB305‐His fusion protein. Promoter fragments of *OsIPT8*, *OsCKX2*, and *OsNAP* were used as 5′‐biotin‐labeled probes (hot probe), and unlabeled fragments were included as competitors (cold probe, 300‐fold molar excess). Red arrows and asterisks indicate shifted DNA–protein complexes and non‐specific bands, respectively.

To identify the *cis*‐elements recognized by OsMYB305 TF, the promoter regions enriched by ChIP‐qPCR were subjected to motif discovery analysis using the Multiple Em for Motif Elicitation (MEME) suite (https://meme‐suite.org/meme/). This analysis identified three candidate motifs (Motif 1–3) (Fig. 5a). While the *pOsIPT8*‐P2 and *pOsNAP*‐P2 regions contained all three motifs, the *pOsCKX2*‐P1 region harbored only Motifs 1 and 3 (Fig. 5b). To validate binding specificity, we performed EMSA using purified OsMYB305‐His protein and probes containing each candidate motif (Fig. S12). DNA–protein complexes were formed with probes containing Motif 3, but not with those containing Motif 1 or 2 (Fig. 5c). These results indicated that OsMYB305 specifically recognizes Motif 3 within the promoters of *OsIPT8*, *OsCKX2*, and *OsNAP*, providing a molecular basis for its direct transcriptional regulation of these target genes.

### Natural variation in *OsMYB305* is associated with subspecies‐specific leaf senescence

**Fig. 6:**
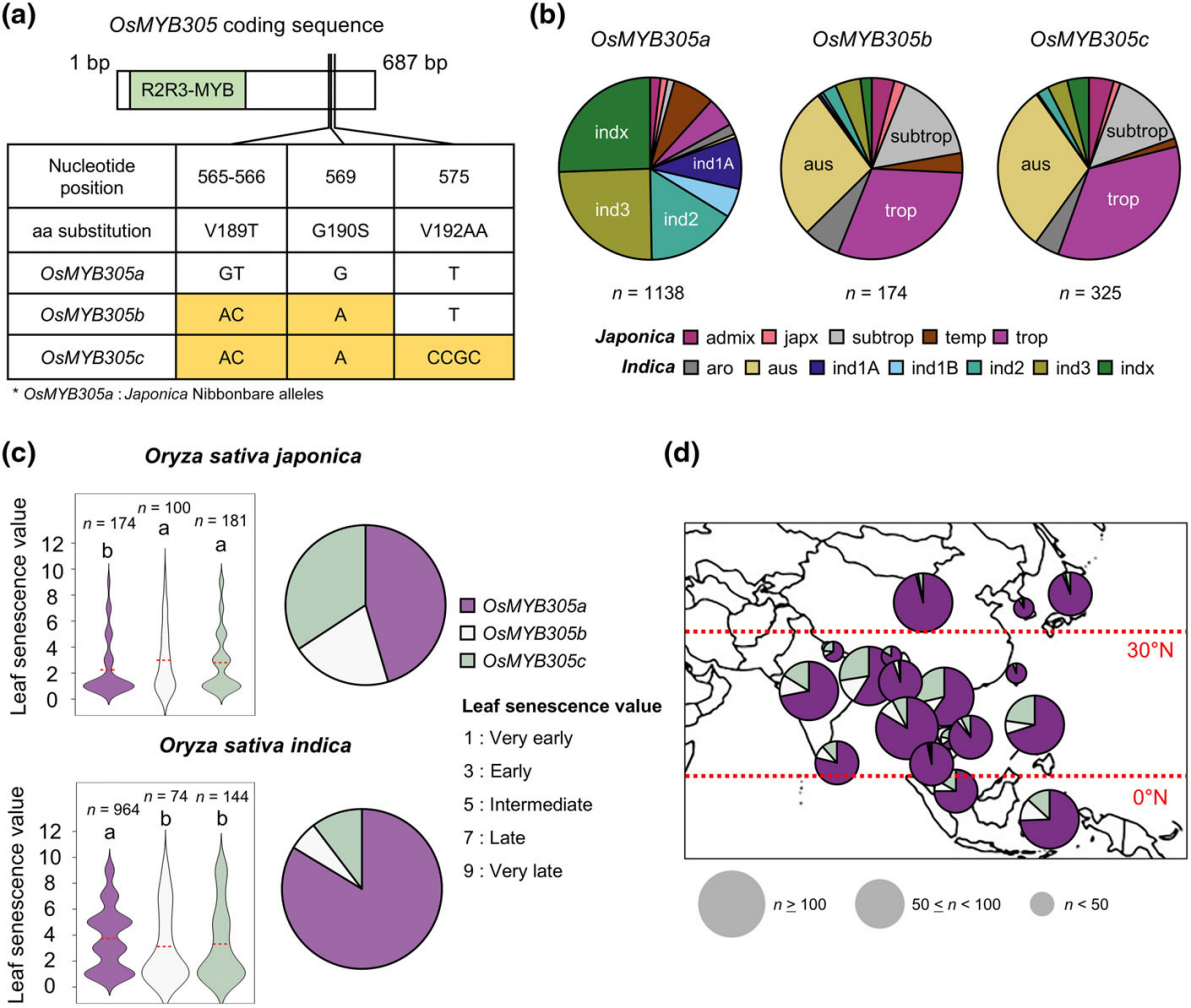
Natural variation of *OsMYB305* alleles in cultivated rice varieties. (a) Analysis of nonsynonymous single‐nucleotide polymorphisms (SNPs) in *OsMYB305* alleles among rice accessions from the International Rice Genebank Collection Information System (IRGCIS). Based on SNP variation, cultivated rice accessions were classified into three haplotypes: *OsMYB305*a, *OsMYB305b*, and *OsMYB305*c. SNP positions are indicated relative to the translation start site of the *OsMYB305* coding sequence, and polymorphic nucleotides are highlighted in yellow. (b) Distribution of cultivated rice subpopulations for *OsMYB305* haplotypes. Accessions were categorized into 12 subpopulations and further grouped into *japonica*‐type (admix, japx, subtrop, temp, and trop) or *indica*‐type (aro, aus, ind1A, ind1B, ind2, ind3, and indx). (c) Violin plots show leaf senescence values of *japonica*‐ and *indica*‐type accessions harboring different *OsMYB305* haplotypes. Leaf senescence values were obtained from IRGCIS, where lower and higher values indicate earlier and later leaf senescence, respectively (scale range: 1–9). Red dashed horizontal lines indicate the mean value for each group. Statistical analyses were performed using one‐way ANOVA followed by Tukey's honestly significant difference test and different letters indicate significant differences among groups within the same treatment (*P* < 0.05). (d) Geographic distribution of cultivated rice accessions carrying different *OsMYB305* alleles across Asia. Circle size means the number of accessions.

We surveyed natural variation in the *OsMYB305* coding region among cultivated rice (*O. sativa*) accessions using the IRGCIS database (Mansueto *et al*., 2017). Nonsynonymous SNPs classified 1637 *O. sativa* accessions into three haplotypes (*OsMYB305a*, *OsMYB305b*, and *OsMYB305c*) (Fig. 6a; Table S2). The Nipponbare (*O. sativa japonica*) genome was used as the reference, and the parental cultivar Dongjin carries the *OsMYB305a* allele. We further examined sequence variation in wild rice (*O. rufipogon*), the ancestral species of cultivated rice. A nonsynonymous SNP located within the R2R3‐MYB DNA‐binding domain was identified in *O. rufipogon*, which was distinct from the polymorphisms detected in *O. sativa* (Fig. S13; Table S3). The WT used in this study carried the *OsMYB305a* allele. 85% of *indica* varieties harbored the *OsMYB305a* allele, whereas more than half of the *OsMYB305b* and *OsMYB305c* alleles were found in *japonica* varieties (Fig. 6b). To investigate the association between *OsMYB305* haplotypes and leaf senescence, we analyzed leaf senescence values corresponding to each accession. Among *Japonica* accessions, varieties carrying the *OsMYB305b* and *OsMYB305c* alleles exhibited late leaf senescence values compared with those carrying the *OsMYB305a*. By contrast, among *indica* accessions, varieties harboring the *OsMYB305b* and *OsMYB305c* alleles showed slightly earlier leaf senescence values relative to those harboring *OsMYB305a* (Fig. 6c; Tables S4, S5). Geographical distribution analysis further revealed that most rice varieties cultivated in high‐latitude regions (Republic of Korea, Japan, and China) predominantly carried the *OsMYB305a* allele, suggesting potential selection and regional adaptation associated with *OsMYB305* haplotypes (Fig. 6d; Table S6).

### Natural variation in the *OsIPT8* promoter affects OsMYB305 binding to the *cis*‐element

**Fig. 7:**
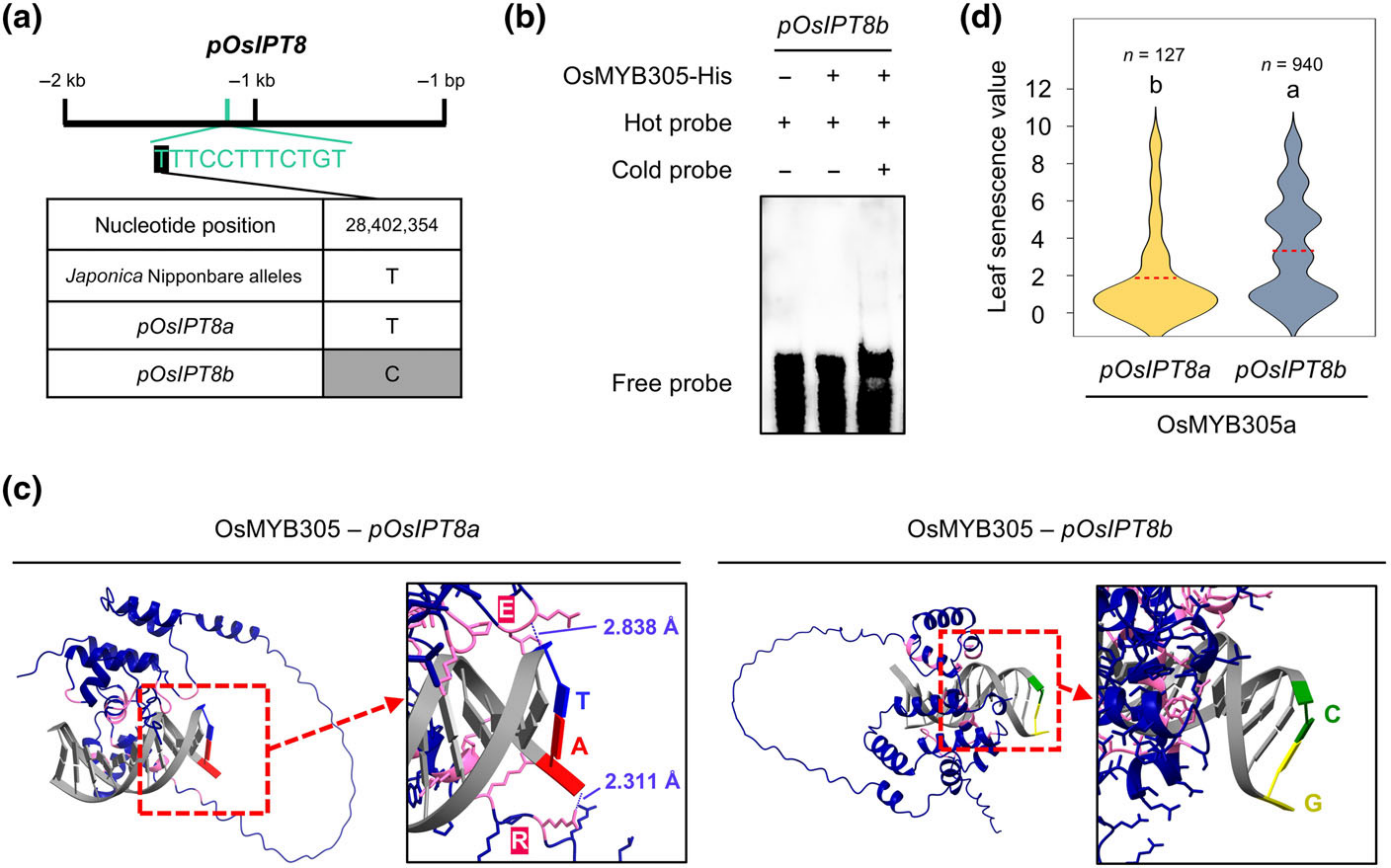
Natural variation in the *cis*‐element of the *pOsIPT8* promoter affects OsMYB305 binding affinity. (a) Analysis of single‐nucleotide polymorphisms (SNPs) in the *cis*‐element of *OsIPT8* promoter (*pOsIPT8*) among 3000 rice accessions from the International Rice Genebank Collection Information System (IRGCIS). The light green vertical line and letters indicate the *cis*‐element recognized by OsMYB305. A SNP is highlighted in black. (b) Electrophoretic mobility shift assay was performed using purified OsMYB305‐His fusion protein. Promoter fragments of *OsIPT8a* and *OsIPT8b* were used as 5′‐biotin‐labeled probes (hot probes), and unlabeled fragments were included as competitors (cold probe, 300‐fold molar excess). (c) Predicted protein–DNA complex structures between OsMYB305 and *pOsIPT8* haplotypes generated using AlphaFold3. Gray and blue represent the *cis*‐element and OsMYB305 protein structure, respectively. Protein residues within 4 Å of DNA are shown in pink. SNP nucleotides are highlighted (T, Thymine; A, Adenosine; C, cytosine; G, guanine). Red shadings indicate protein residues (E, glutamate; R, arginine) positioned at 2.838 Å and 2.311 Å from the *cis*‐element. (d) Violin plots showed leaf senescence values associated with *pOsIPT8a* and *pOsIPT8b* haplotypes in rice cultivars carrying *OsMYB305a*. Leaf senescence values were obtained from IRGCIS, where lower and higher values indicate earlier and later leaf senescence, respectively (scale range: 1–9). Red dashed horizontal lines indicate the mean value for each group. Statistical analyses were performed using One‐way ANOVA followed by Tukey's honestly significant difference test, and different letters indicate significant differences among groups within the same treatment (*P* < 0.05).

To investigate whether natural variation in the *cis*‐element within the promoter regions affects the binding affinity of OsMYB305, we surveyed SNPs within the 2‐kb upstream regions of *OsIPT8*, *OsCKX2*, and *OsNAP* (Tables S7–S9). Among the identified SNPs, a T to C substitution within Motif 3 in the *OsIPT8* promoter divided the accessions into two haplotypes, *pOsIPT8*a and *pOsIPT8b* (Fig. 7a). EMSA revealed that the OsMYB305‐His protein failed to bind to the *pOsIPT8b* promoter (Fig. 7b), indicating that this nucleotide substitution disrupts OsMYB305 binding affinity to the *cis*‐element. To further explore the structural basis of this interaction, we predicted protein–DNA complex models of OsMYB305 bound to the *cis*‐element using AlphaFold 3. The MYB repeat domain of OsMYB305 was positioned in close proximity to the *cis*‐element with low PAE values. In particular, glutamate (E) and arginine (R) residues of OsMYB305 were predicted to be positioned 2.838 Å and 2.311 Å from thymine and cytosine bases, respectively (Figs 7c, S14). However, the C‐G substitution in the *pOsIPT8b* haplotype altered the spatial positioning of these residues to the *cis*‐element (Fig. 7c). We further analyzed leaf senescence values associated with *pOsIPT8* haplotypes in rice accessions carrying the *OsMYB305a* background. Accessions harboring *pOsIPT8a* exhibited earlier leaf senescence than those carrying *pOsIPT8b* (Fig. 7d). These results indicate that the SNP in the *pOsIPT8b* promoter disrupts OsMYB305 binding affinity to the *cis*‐element, thereby contributing to delayed leaf senescence in rice.

## Discussion

### *OsMYB305* mediates antagonistic regulation between CK and ABA signaling during leaf senescence

**Fig. 8:**
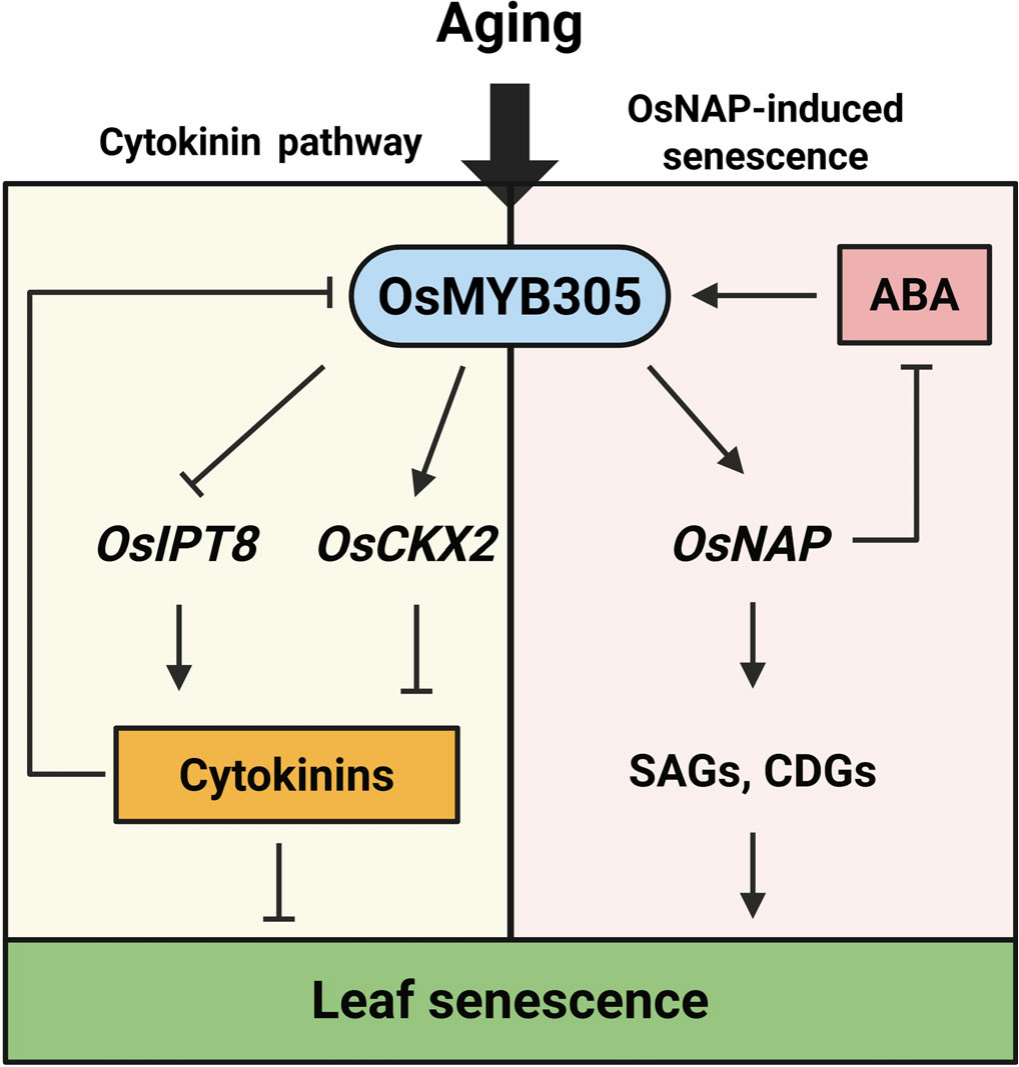
Proposed regulatory model of *OsMYB305* in leaf senescence. *OsMYB305* reduces endogenous cytokinins (CK) levels by repressing CK biosynthesis and promoting CK catabolism. In addition, *OsMYB305* activates *OsNAP* and *OsNAP*‐regulated Chl degradation genes and senescence‐associated genes. Positive and negative regulatory relationships are indicated by arrows and flat‐ended lines, respectively. This figure was created in BioRender (BioRender.com/1mrx286).

Leaf senescence is the final stage of leaf development and is tightly regulated by phytohormones (Distelfeld *et al*., 2014; Kuai *et al*., 2018). Among these hormones, CKs and ABA act antagonistically to control the initiation and progression of senescence. In this study, we identified OsMYB305 as a key regulator of leaf senescence that integrates CK homeostasis and ABA signaling. During leaf senescence, the expression patterns of *OsCKX2* and *OsNAP* gradually increased, closely mirroring that of *OsMYB305*, whereas *OsIPT8* expression showed an opposite trend (Fig. 4a–c). These coordinated transcriptional changes suggest that OsMYB305 contributes to the initiation of leaf senescence by promoting CK depletion and activating OsNAP‐dependent senescence programs. In rice, *OsNAP*, the ortholog of *AtNAP*, is similarly upregulated during senescence or by ABA treatment. OsNAP promotes leaf senescence by activating CDGs and SAGs and also contributes to abiotic stress tolerance through modulation of ABA signaling (Chen *et al*., 2014). In addition, OsNAP suppresses ABA biosynthesis through a feedback regulatory mechanism that differs from the regulatory mode of *AtNAP* (Liang *et al*., 2014). Interestingly, although *osmyb305* mutants displayed reduced ABA signaling (Fig. S8), they accumulated higher endogenous ABA levels accompanied by increased expression of ABA biosynthetic genes (Fig. S7). Given that OsMYB305 activates the *OsNAP*‐mediated senescence pathway, this discrepancy can be explained by a feedback regulation of ABA biosynthesis mediated, at least in part, by *OsNAP* (Fig. 4h; Liang *et al*., 2014). Collectively, *OsMYB305* coordinates age‐dependent leaf senescence in rice through two regulatory pathways. First, OsMYB305 modulates CK homeostasis by repressing CK biosynthesis while promoting CK degradation. Second, OsMYB305 facilitates OsNAP‐dependent senescence progression, leading to the activation of CDGs and SAGs (Fig. 8).

### A signal‐dependent model for OsMYB305 transcriptional duality

OsMYB305 exhibits dual transcriptional regulatory activity, functioning as both an activator and a repressor (Fig. S10). Such transcriptional duality arises through either context‐dependent regulation, determined by promoter architecture and *cis*‐elements, or signal‐dependent regulation, mediated by hormonal cues and PTMs, including phosphorylation and SUMOylation (Tong *et al*., 1996; Boyle & Despres, 2010; Dong & Guertin, 2026). OsMYB305 appears to recognize shared *cis*‐elements in the promoters of both activated and repressed target genes (Fig. 5), indicating that promoter‐dependent mechanisms alone are insufficient to account for its dual functionality. Instead, these findings support a signal‐dependent regulatory model, consistent with *in silico* predictions identifying putative PTM sites within OsMYB305 (Fig. S11). Consistent with this model, Arabidopsis immune regulator NONEXPRESSOR OF PATHOGENESIS‐RELATED GENES 1 (NPR1), which undergoes a SUMOylation‐dependent functional switch in response to SA, thereby altering its transcriptional activity toward downstream gene *PR1* (Spoel *et al*., 2009). Similarly, in the Arabidopsis brassinosteroid signaling pathway, BRASSINAZOLE‐RESISTANT 1 (BZR1)/BRI1‐EMS‐SUPPRESSOR 1 (BES1) are regulated through reversible phosphorylation, and brassinosteroid‐induced dephosphorylation converts them into potent activators (Li *et al*., 2018). Although the molecular basis of OsMYB305 dual functionality remains to be resolved, our results suggest that PTM‐mediated switching may represent a key mechanism regulating its transcriptional activity.

### OsMYB305 recognizes a noncanonical *cis*‐element distinct from previously defined MYB motifs

TFs act as the upstream regulators of complex gene networks by directly binding to the promoter regions of their target genes. The R2R3‐MYB DNA‐binding domain, composed of one to four MYB repeats, adopts a helix–turn–helix structure that facilitates sequence‐specific DNA recognition (Prouse & Campbell, 2012). This domain typically recognizes the MYB‐core motif ([C/T]NGTT[G/T]), which can be subdivided into type I (CNGTT[G/A]) and type II (TNGTT[G/A]) elements, as well as AC‐rich elements, all of which serve as key *cis*‐regulatory sequences governing transcriptional activity in plants (Prouse & Campbell, 2012; Millard *et al*., 2019). Large‐scale Y1H analyses have shown that Arabidopsis R2R3‐MYB TFs preferentially bind to the MYB‐core type I motifs (Kelemen *et al*., 2015). Citrus (*Citrus unshiu* Marc.) CiMYB42 TF specifically recognizes the TTGTTG sequence (Zhang *et al*., 2020). In addition, AC‐rich elements are recognized by MYB305 TF in ornamental tobacco (*Nicotiana langsdorffii* x *N. sanderae*) (Liu & Thornburg, 2012). Similarly, *Pinus taeda* MYB1 (PtMYB1) and *Eucalyptus grandis* MYB2 (*EgMYB2*) bind to the AC‐rich elements to promote lignified cell differentiation (Patzlaff *et al*., 2003; Goicoechea *et al*., 2005).

However, the extensive functional diversification of MYB TFs has resulted in substantial variation in their cognate *cis*‐regulatory motifs across plant species. For instance, apple (*Malus domestica*) MYB10 (MdMYB10) binds to a noncanonical ACTGGTAGCTATT sequence (Xie *et al*., 2010). Tobacco (*N. tabacum*) NtMYB305a recognizes a GAG region composing a G‐box, an AT‐rich motif, and a GCC‐box‐like element (Bian *et al*., 2022). Notably, NtMYB305a TF also interacts with a 30‐bp AT‐rich motif lacking G/C base, which differs from canonical AC‐rich elements (Prouse & Campbell, 2012; Bian *et al*., 2022). A previous report identified the TTHGGY motif (H indicates A/T/G, Y indicates T/C) as a binding sequence recognized by OsMYB8/OsMYB305 (Gou *et al*., 2024a). In this study, we found that OsMYB305 does not bind to ACC[C/G]CT and GCC[G/A]GG motifs, which resemble AC‐rich and GCC box‐like elements, respectively (Fig. 5). The TTHGGY motif was absent from the promoter region of *OsIPT8*, *OsCKX2*, and *OsNAP*. Instead, OsMYB305 specifically recognized the TG[T/A][T/G/C]TTTC[C/T]TTT motif, which is distinct from previously defined MYB‐binding sequences (Figs 5, S9). Moreover, a single‐nucleotide substitution disrupted OsMYB305 binding affinity to this motif, indicating that natural variation within the *cis*‐element can alter OsMYB305‐DNA interactions (Fig. 7). Plant TFs frequently regulate diverse biological pathways by recognizing multiple *cis*‐elements rather than relying on a single conserved motif (Franco‐Zorrilla *et al*., 2014; Xie *et al*., 2019). Our findings indicate that MYB TF target specificity is not governed by a single canonical binding sequence, but rather reflects a diverse repertoire of *cis*‐elements across MYB family members, enabling fine‐tuned transcriptional regulation within complex gene regulatory networks.

### Subspecies‐specific effects of the *OsMYB305a* haplotype on leaf senescence

In this study, the delayed leaf senescence phenotype of the *osmyb305* was more pronounced than the accelerated leaf senescence observed in *OsMYM305*‐OEs (Figs 2, S3). All transgenic materials were generated in *japonica* cultivar Dongjin, providing a uniform genetic background for phenotypic comparison. Our haplotype analysis further revealed that the *OsMYB305a* allele in *japonica* accessions is associated with relatively earlier senescence compared with the same allele in *indica* accessions (Fig. 6c), indicating that the functional output of *OsMYB305a* is modulated by subspecies‐specific genetic contexts. This background‐dependent effect provides a plausible explanation for the asymmetric phenotypes observed between loss‐of‐function and overexpression lines. In the relatively fast‐senescing Dongjin background, loss of *OsMYB305* function leads to a substantial delay in senescence, whereas overexpression results in moderate acceleration of senescence, potentially reflecting physiological constraints on further advancing an already rapid senescence program. Together, these findings suggest that subspecies divergence shapes the phenotypic impact of *OsMYB305a* on leaf senescence.

### Adaptive selection of *OsMYB305* haplotypes under high‐latitude environments

In the temperate and high‐latitude rice fields such as Korea, Japan, and northeastern China, nitrogen uptake frequently declines during the grain‐filling period due to decreasing soil temperatures and reduced root metabolic activity (Sun *et al*., 2025). Although prolonged photosynthetic activity during grain filling can enhance leaf carbon assimilation, extended canopy longevity does not necessarily translate into a yield advantage under conditions of limited nitrogen uptake (Thomas & Ougham, 2014). In these scenarios, the late senescence values associated with *OsMYB305b* and *OsMYB305c* haplotypes, which resemble *osmyb305* loss‐of‐function mutants, could be disadvantageous under temperate and high‐latitude regions where nitrogen remobilization is critical for stable grain filling (Figs 2, 6c). Therefore, regional environmental constraints may have contributed to the preferential retention or artificial selection of the *OsMYB305a* haplotype in temperate and high‐latitude rice‐growing regions (Fig. 6c,d).

Interestingly, the SNP identified in wild rice (*O. rufipogon*) was absent in cultivated rice (*O. sativa*), whereas the distinct *OsMYB305* SNPs detected in cultivated rice were not observed in wild rice (Fig. S13). The cultivation‐specific polymorphisms likely arose through *de novo* mutations after the onset of domestication and were subsequently retained under artificial and regional selection. Thus, we propose that the domestication‐derived SNPs in *OsMYB305* may represent adaptive variants that confer selective advantages to *japonica* rice, particularly in temperate and high‐latitude regions.

## Competing interests

None declared.

## Author contributions

N‐CP and KK conceived and supervised the project. BK and Jinah Kim designed the research and performed all of the experiments with the assistance of YS, Jinku Kang, HY and S‐HC. BK, N‐CP and KK analyzed the data and wrote the manuscript. All authors have read and approved the published version of the manuscript. BK and Jinah Kim contributed equally to this work.

## Disclaimer

The New Phytologist Foundation remains neutral with regard to jurisdictional claims in maps and in any institutional affiliations.

## Supporting information


**Fig. S1** Spatial expression patterns of *OsMYB305*.
**Fig. S2** Identification of *osmyb305* mutants and *OsMYB305* overexpression lines.
**Fig. S3** Overexpression of *OsMYB305* accelerates leaf senescence.
**Fig. S4** Flowering time of *osmyb305* mutants.
**Fig. S5** Expression of senescence‐associated genes in *osmyb305* mutants.
**Fig. S6** Agronomic traits of *osmyb305* mutants.
**Fig. S7** Altered endogenous abscisic acid (ABA) contents and ABA‐related genes in *osmyb305* mutants.
**Fig. S8** *osmyb305* mutants are insensitive to abscisic acid.
**Fig. S9** OsMYB305 does not bind to the promoter of *OsIPT5, OsCKX4, OsCKX9*, or *OsCKX11*.
**Fig. S10** Transrepression and transactivation activities of OsMYB305.
**Fig. S11** Prediction of phosphorylation and SUMOylation site of OsMYB305 *in silico*.
**Fig. S12** Purification of recombinant OsMYB305‐His protein.
**Fig. S13** Natural variation of *OrMYB305* in wild rice *Oryza rufipogon*.
**Fig. S14** AlphaFold 3‐predicted structures of the OsMYB305–*pOsIPT8* haplotype protein–DNA complexes.

**Table S1** Primers used in this study.
**Table S2** List of the nonsynonymous single‐nucleotide polymorphisms at *OsMYB305* in rice varieties in the International Rice Genebank Collection Information System.
**Table S3** List of the nonsynonymous single‐nucleotide polymorphisms at *OrMYB30*5 in 446 *Oryza rufipogon* accessions.
**Table S4** List of the nonsynonymous single‐nucleotide polymorphisms at *OsMYB305* in *Oryza sativa japoninca* varieties.
**Table S5** List of the nonsynonymous single‐nucleotide polymorphisms at *OsMYB305* in *Oryza sativa indica* varieties.
**Table S6** List of the varieties used for geographical distribution analysis.
**Table S7** List of the single‐nucleotide polymorphisms at the promoter of *OsIPT8* in rice varieties in the International Rice Genebank Collection Information System.
**Table S8** List of the single‐nucleotide polymorphisms at the promoter of *OsCKX2* in rice varieties in the International Rice Genebank Collection Information System.
**Table S9** List of the single‐nucleotide polymorphisms at the promoter of *OsNAP* in rice varieties in the International Rice Genebank Collection Information System.
Please note: Wiley is not responsible for the content or functionality of any Supporting Information supplied by the authors. Any queries (other than missing material) should be directed to the *New Phytologist* Central Office.

## Data Availability

The data that support the findings of this study are available in the supplementary material of this article (Supporting Information S1–S14 and Tables S1–S9). Sequence data generated in this study can be found in the National Center for Biotechnology Information (NCBI) Database and Resource (https://www.ncbi.nlm.nih.gov/) under the following accession nos. (LOC_): *OsMYB305* (Os01g45090), *OsNAP* (Os03g21060), *Osl57* (Os02g57260), *OsSGR* (Os09g36200), *OsNYC3* (Os06g24730), *OsNCED1* (Os02g47510), *OsNCED2* (Os12g24800), *OsNCED3* (Os03g44380), *OsABA8ox1* (Os02g47470), *OsABA8ox2* (Os08g36860), *OsABA8ox3* (Os09g28390), *OsIPT5* (Os07g11050), *OsIPT8* (Os01g49390), *OsCKX2* (Os01g10110), *OsCKX4* (Os01g71310), *OsCKX9* (Os05g31040), *OsCKX11* (Os08g35860), *OsABF2* (Os06g10880), *OsABF4* (Os09g28310), *OsABI5* (Os01g64730), Os*UBQ5* (Os01g22490), *Actin* (Os03g50885).
