# Supplementary material for: Natural variation in OsMYB305 downregulating cytokinin‐mediated inhibition of leaf senescence contributes to regional adaptation in rice
Source: New Phytol. 2026 Jun 4;251(4):1873–88. doi: 10.1111/nph.71335 (PMC13373818; doi:10.1111/nph.71335)
Supplement: Supplementary file 1 — Fig. S1 Spatial expression patterns of OsMYB305. Fig. S2 Identification of osmyb305 mutants and OsMYB305 overexpression lines. Fig. S3 Overexpression of OsMYB305 accelerates leaf senescence. Fig. S4 Flowering time of osmyb305 mutants. Fig. S5 Expression of senescence‐associated genes in osmyb305 mutants. Fig. S6 Agronomic traits of osmyb305 mutants. Fig. S7 Altered endogenous abscisic acid (ABA) contents and ABA‐related genes in osmyb305 mutants. Fig. S8 osmyb305 mutants are insensitive to abscisic acid. Fig. S9 OsMYB305 does not bind to the promoter of OsIPT5, OsCKX4, OsCKX9, or OsCKX11. Fig. S10 Transrepression and transactivation activities of OsMYB305. Fig. S11 Prediction of phosphorylation and SUMOylation site of OsMYB305 in silico. Fig. S12 Purification of recombinant OsMYB305‐His protein. Fig. S13 Natural variation of OrMYB305 in wild rice Oryza rufipogon. Fig. S14 AlphaFold 3‐predicted structures of the OsMYB305–pOsIPT8 haplotype protein–DNA complexes. [file NPH-251-1873-s001.pdf]

New Phytologist Supporting Information

Article title: Natural variation in OsMYB305 downregulating cytokinin-mediated inhibition of leaf senescence contributes to regional adaptation in rice

Authors: Boyeong Kim, Jinah Kim, Yejin Shim, Jinku Kang, Hyeryung Yoon, Sung-Hwan Cho, Nam-Chon Paek, Kiyoon Kang

Article acceptance date: 16 May 2026

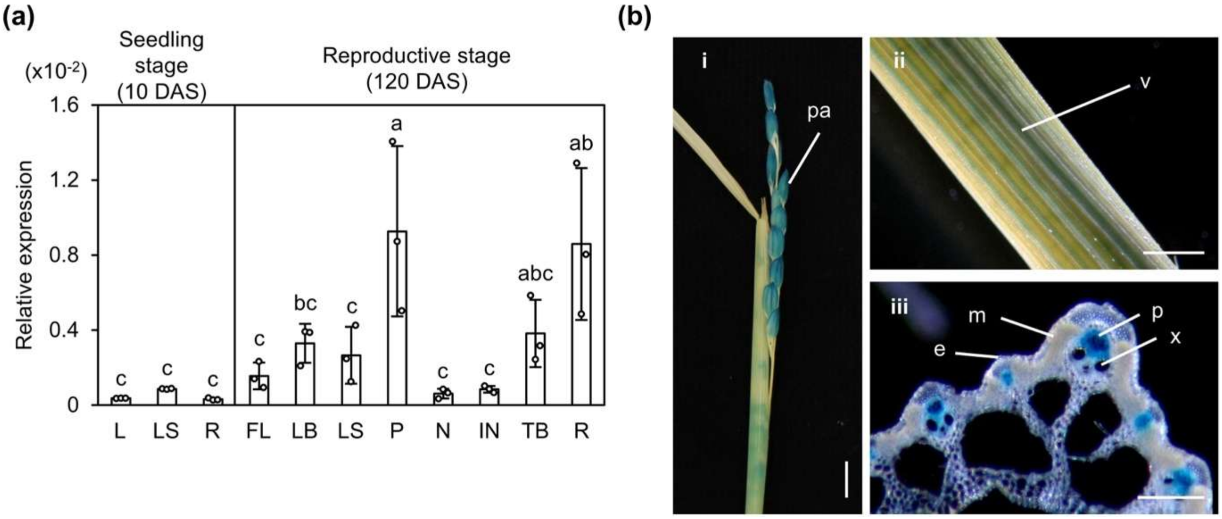

Fig. S1 Spatial expression patterns of *OsMYB305*. (**a**) Rice WT plants were grown in a growth chamber for 10 days after sowing (DAS) under long-day conditions or grown in a paddy field for 120 DAS under natural long day conditions. L, leaf; LS, leaf sheath; R, root; FL, flag leaf; LB, leaf blade; P, panicle; N, node; IN, internode; TB, tiller base. Data are presented as means ± SD (n = 3 biological replicates), with each replicate consisting of four plants per genotype. Statistical analyses were performed using one-way ANOVA followed by Tukey's HSD test, with different letters indicating significant differences among groups within the same treatment ($p < 0.05$). (**b**) Histochemical β-glucuronidase (GUS) staining of transgenic rice plants expressing *pOsMYB305*::GUS. (i) a panicle at 5 DAH, (ii) a leaf sheath at 120 DAS, and (iii) a cross-section of the leaf sheath. Scale bars = 1 cm (i), 1 mm (ii), and 200 μm (iii). pa, panicle; v, vein; e, epidermis; m, mesophyll cell; x, xylem; p, phloem.

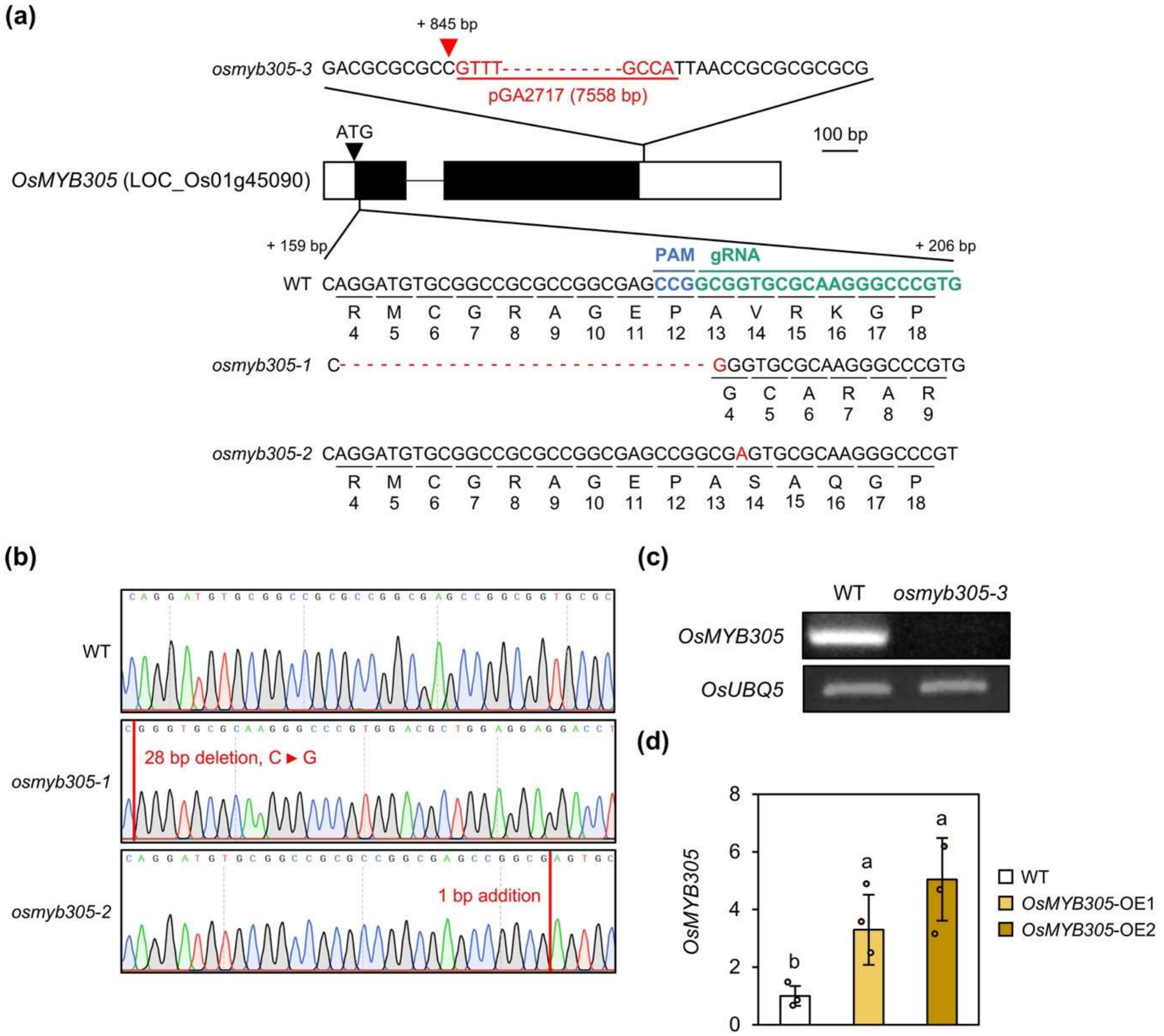

Fig. S2 Identification of *osmyb305* mutants and *OsMYB305* overexpression lines. (**a**) Schematic representation of the rice wild-type (WT) and *osmyb305* mutants (*osmyb305-1, osmyb305-2*, and *osmyb305-3*). Black and white boxes represent exons and untranslated regions of *OsMYB305*, respectively. Green and blue lowercase letters represent the single guide RNA (sgRNA) target sequence and the protospacer adjacent motif (PAM), respectively. Red letters and dots indicate the positions of T-DNA insertion or CRISPR/Cas9-mediated mutations. Amino acids encoded by the nucleotide triplets are shown below the sequences. Arabic numerals indicate amino acid positions relative to the translation start site. (**b**) Sanger sequencing chromatograms of the parental line WT, *osmyb305-1* and *osmyb305-2*. Red vertical lines represent the predicted double-stranded break (DSB) site. (**c**,**d**) Rice plants were grown in a paddy field under natural long day conditions. (**c**) Semi-quantitative RT-PCR analysis of

OsMYB305 transcripts in leaves of *osmyb305-3* and its parental line. Rice *UBIQUITIN5* (*OsUBQ5*) was used as an internal control. (**d**) Relative transcript levels of *OsMYB305* in WT and *OsMYB305* overexpression lines (*OsMYB305*-OE1 and *OsMYB305*-OE2) were determined by RT-qPCR and normalized to *OsUBQ5* using the $2^{-\Delta\Delta CT}$ method. Data are presented as means ± SD (n = 3 biological replicates), with each replicate consisting of four plants per genotype. Statistical analyses were performed using one-way ANOVA followed by Tukey's HSD test, with different letters indicating significant differences among groups within the same treatment ($p < 0.05$).

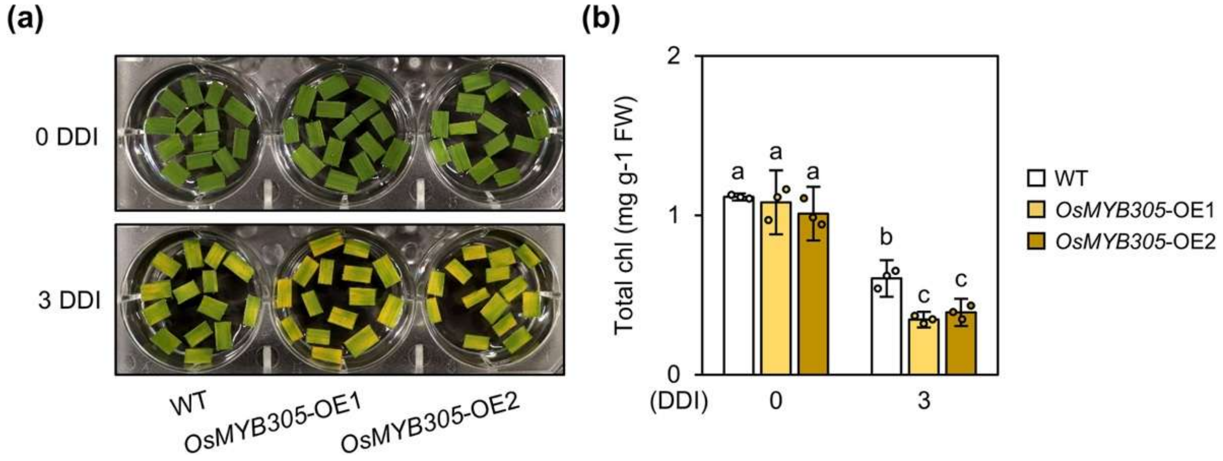

Fig. S3 Overexpression of *OsMYB305* accelerates leaf senescence. (**a-b**) Flag leaves from rice plants grown in a paddy field under natural long day conditions were incubated in 3 mM MES (pH 5.8) under complete darkness at 28 °C. Representative phenotypes (a) and total chlorophyll (Chl) contents in wild-type (WT) and *OsMYB305*-overexpressing lines (*OsMYB305*-OE1 and *OsMYB305*-OE2) (b) were measured after 3 days of dark incubation (DDI). These experiments were independently repeated three times using separate sets of plates, with similar results. Data are presented as means ± SD (n = 3 biological replicates). Statistical analyses were performed using one-way ANOVA followed by Tukey's HSD test, with different letters indicating significant differences among groups within the same treatment ($p < 0.05$).

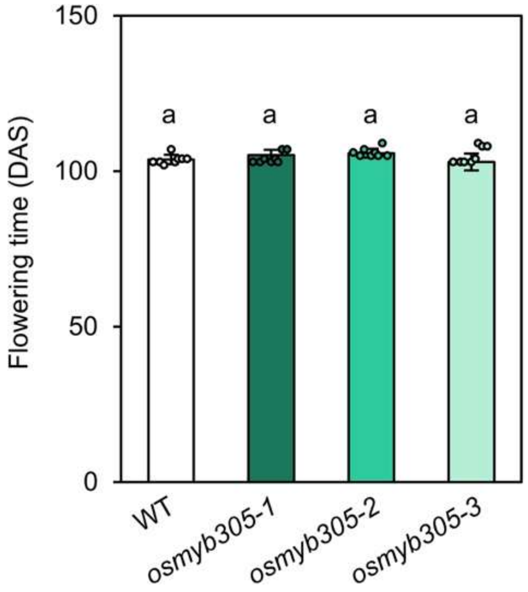

Fig. S4 Flowering time of *osmyb305* mutants. Rice wild-type (WT) and *osmyb305* mutants were grown in a paddy field under natural long day conditions. Means and standard deviations were obtained from eight biological replicates. Data are presented as means ± SD (n = 7 biological replicates). Statistical analyses were performed using one-way ANOVA followed by Tukey's HSD test, with different letters indicating significant differences among groups within the same treatment ($p < 0.05$).

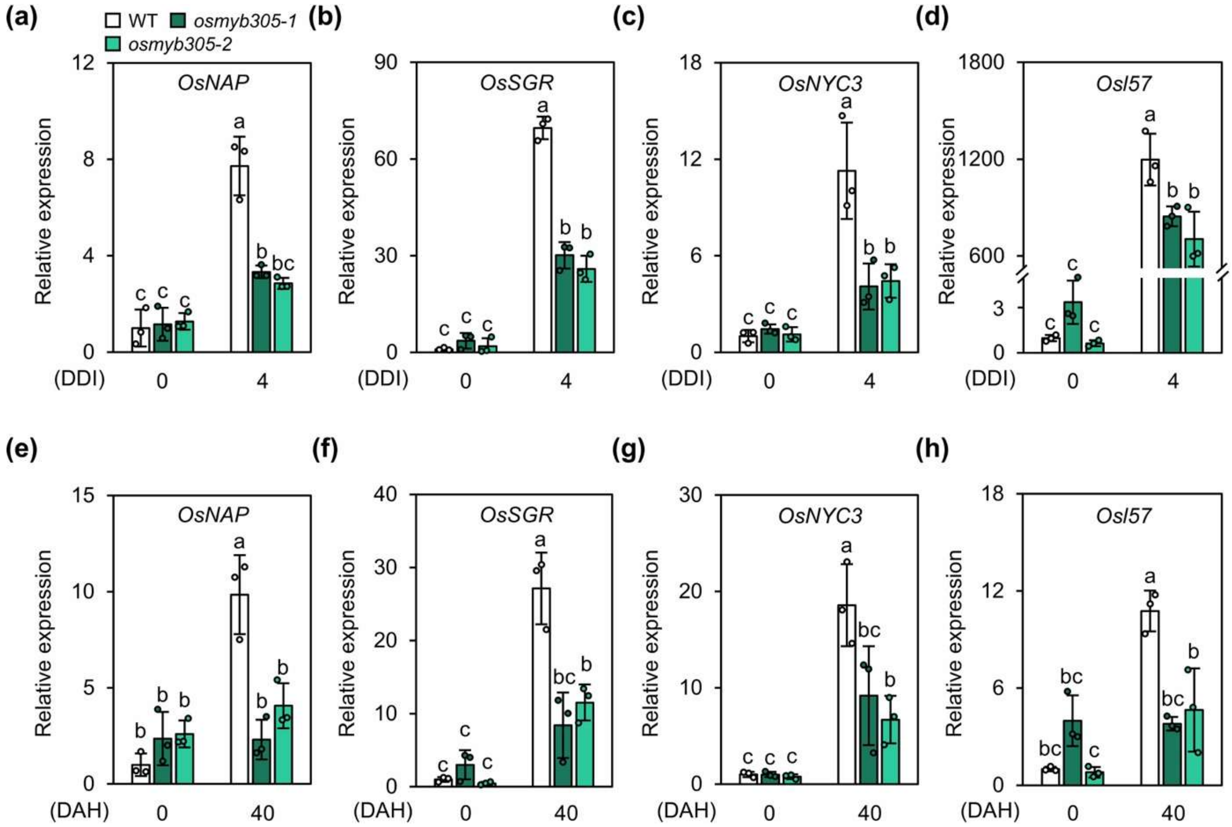

Fig. S5 Expression of senescence-associated genes in *osmyb305* mutants. (**a-h**) Total RNA was extracted from flag leaves of rice wild-type (WT) and *osmyb305* mutants at 0 and 4 days of dark incubation (DDI) (a-d) or 0 and 40 days after heading (DAH) (e-h). Relative transcript levels of *OsNAP*, *OsSGR*, *OsNYC3*, and *OsI57* were determined by reverse transcription-quantitative PCR (RT-qPCR) and normalized to rice *UBIQUITIN5* (*OsUBQ5*) using the $2^{-\Delta\Delta CT}$ method. Data are presented as means ± SD (n = 3 biological replicates), with each replicate consisting of four plants per genotype. Statistical analyses were performed using one-way ANOVA followed by Tukey's HSD test, with different letters indicating significant differences among groups within the same treatment ($p < 0.05$).

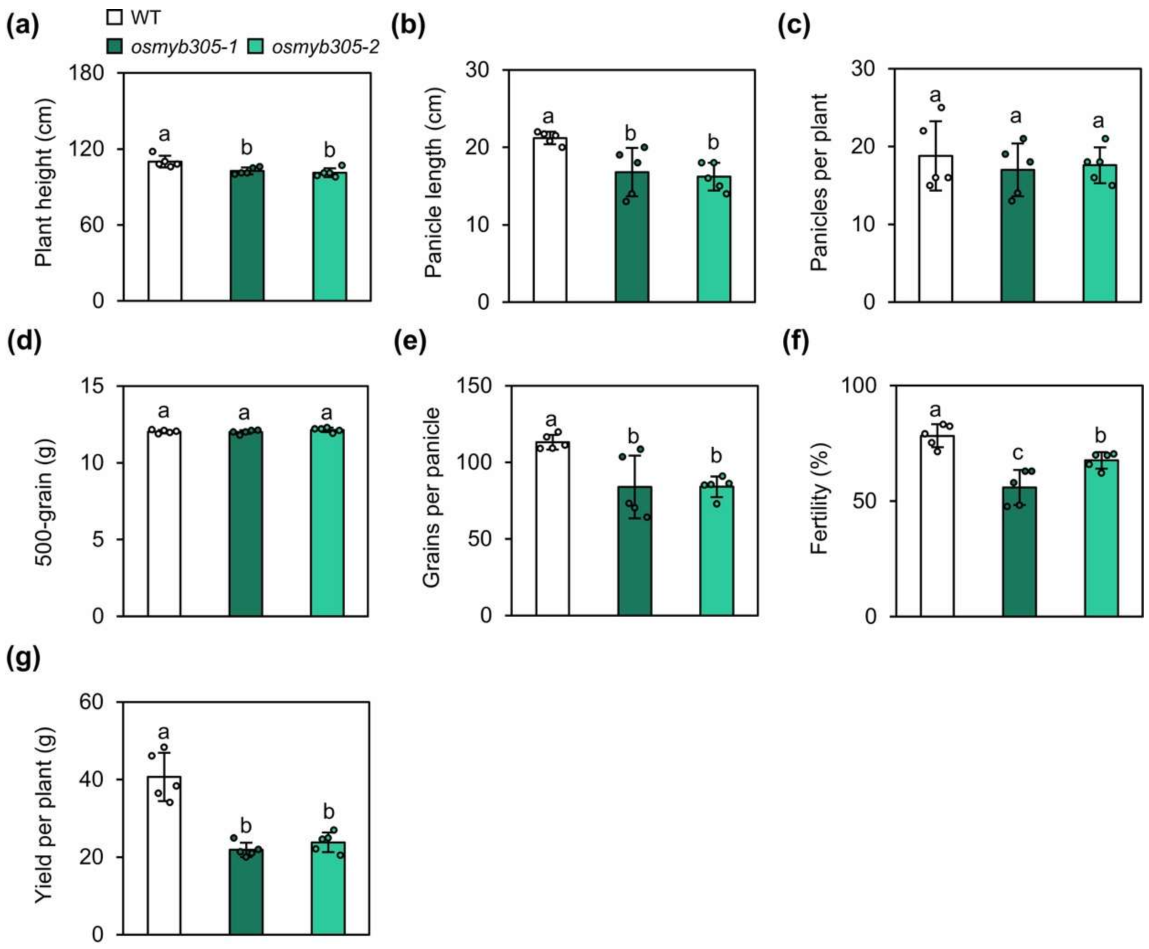

Fig. S6 Agronomic traits of *osmyb305* mutants. (**a-g**) Rice wild-type (WT) and *osmyb305* mutants were cultivated in a paddy field under natural long day conditions. Measurements of plant height (a), panicle length (b), panicles per plant (c), 500-grain weight (d), grains per panicle (e), fertility (f), and yield per plant (g). Data are presented as means ± SD (n = 5 biological replicates). Statistical analyses were performed using one-way ANOVA followed by Tukey's HSD test, with different letters indicating significant differences among groups within the same treatment ($p < 0.05$).

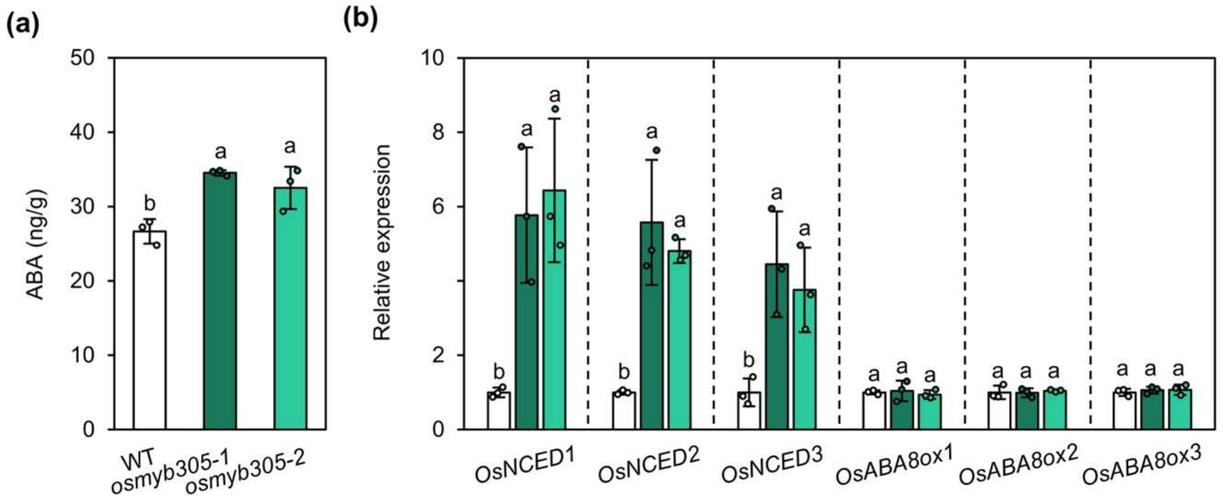

Fig. S7 Altered endogenous ABA contents and ABA-related genes in *osmyb305* mutants. (**a**-**b**) Rice wild-type (WT) and *osmyb305* mutants were grown in a paddy field under natural long day conditions. Quantification of endogenous abscisic acid (ABA) contents (a) and the expression of ABA biosynthetic (*OsNCED1*, *OsNCED2*, and *OsNCED3*) and catabolic (*OsABA8ox1*, *OsABA8ox2*, and *OsABA8ox3*) genes (b) were measured in flag leaves at 30 DAH. Relative transcript levels were determined by reverse transcription-quantitative PCR (RT-qPCR) and normalized to rice *UBIQUITIN5* (*OsUBQ5*) using the $2^{-\Delta\Delta CT}$ method. Data are presented as means ± SD (n = 3 biological replicates), with each replicate consisting of eight (a) or four (b) plants per genotype. Statistical analyses were performed using one-way ANOVA followed by Tukey's HSD test, with different letters indicating significant differences among groups within the same treatment ($p < 0.05$).

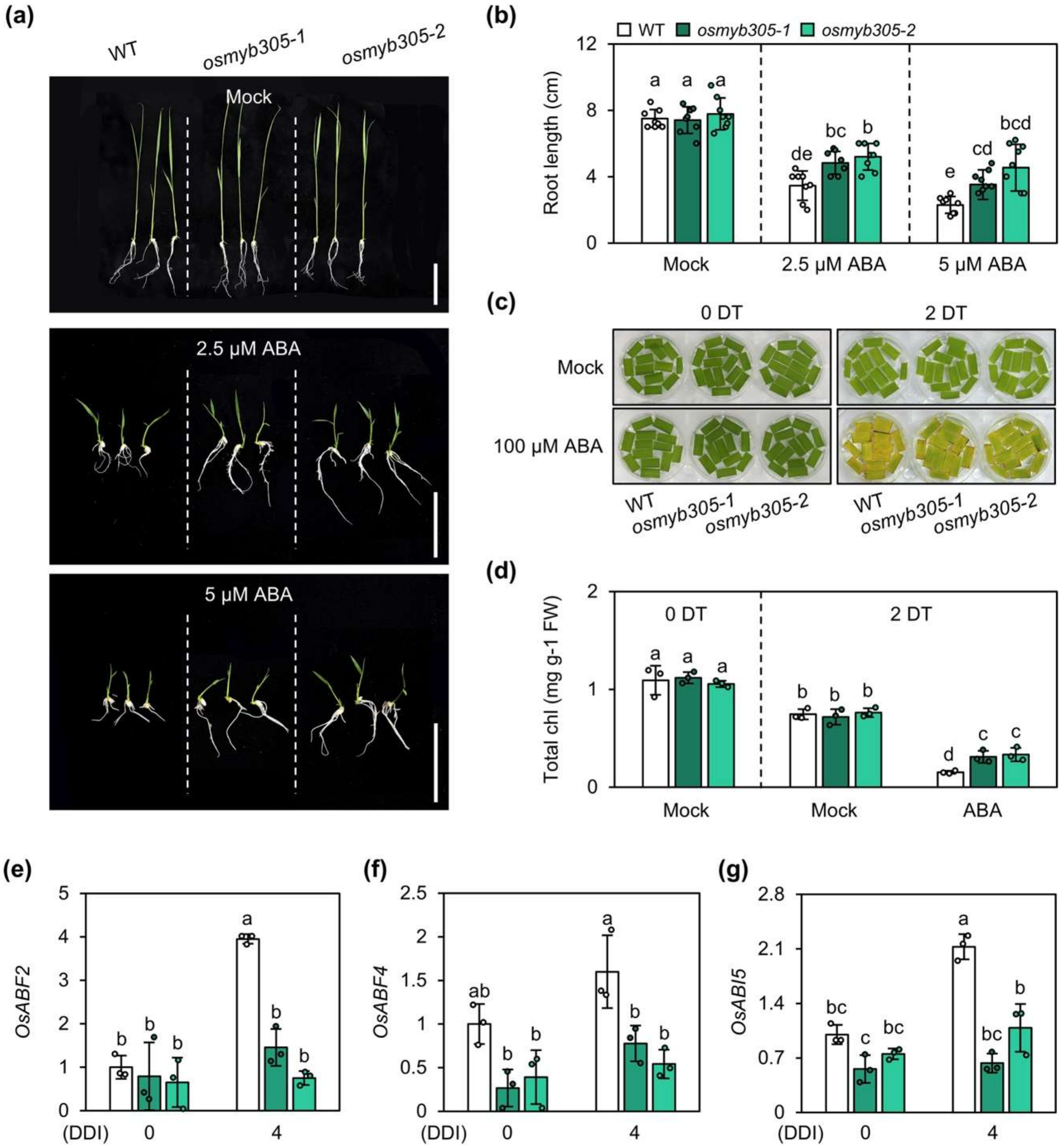

Fig. S8 *osmyb305* mutants are insensitive to abscisic acid (ABA). (**a**,**b**) Rice wild-type (WT) and *osmyb305* mutants were germinated on 1/2 Murashige and Skoog (MS) phytoagar medium for 3 days and subsequently transferred to 1/2 MS phytoagar medium supplemented with 2.5 or 5 μM ABA. Seedlings grown on ABA-free medium served as mock controls. Representative phenotypes (a) and primary root lengths (b) were assessed 7 days after treatment. Scale bar = 5 cm. (**c**,**d**) Detached flag leaves of rice WT and *osmyb305* mutants were incubated in 3 mM

MES (pH 5.8) supplemented with 100 μM ABA. Leaves incubated without ABA served as mock controls. Leaf yellowing phenotypes (c) and total chlorophyll (Chl) contents (d) were investigated at 2 days after treatment (DT). These experiments were independently repeated three times using separate sets of plates, with similar results. (**e-g**) Total RNA was extracted from detached leaves of rice WT and *osmyb305* mutants (*osmyb305-1*, *osmyb305-2*, and *osmyb305-3*) subjected to 0, and 4 days of dark incubation (DDI). Relative transcript levels of ABA signaling genes, *OsABF2* (e), *OsABF4* (f), and *OsABI5* (g) were determined by reverse transcription-quantitative PCR (RT-qPCR) and normalized to rice *UBIQUITIN5* (*OsUBQ5*) using the $2^{-\Delta\Delta CT}$ method. Data are presented as means ± SD (n = 7 (b) and n = 3 (d-g) biological replicates. For (e–g), each biological replicate consisted of four plants per genotype). Statistical analyses were performed using one-way ANOVA followed by Tukey's HSD test, with different letters indicating significant differences among groups within the same treatment ($p < 0.05$).

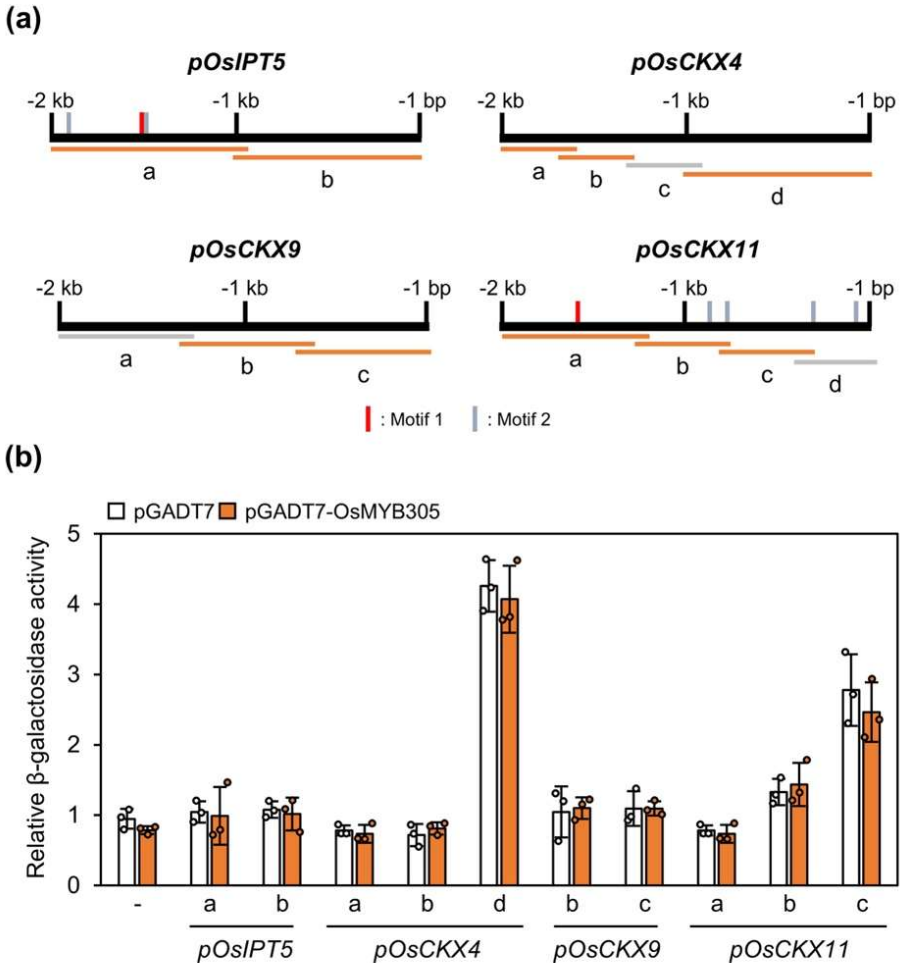

Fig. S9 OsMYB305 does not bind to the promoter of *OsIPT5, OsCKX4, OsCKX9*, or *OsCKX11*. (**a**) Schematic representation of the *OsIPT5*, *OsCKX4*, *OsCKX9*, and *OsCKX11* promoter regions used for yeast one-hybrid (Y1H) assays. Orange horizontal bars depict promoter fragments used for Y1H assays. Gray horizontal bars represent promoter regions that showed self-activation in Y1H assays. (**b**) Y1H assays were determined by measuring β-galactosidase activity using chlorophenol red-β-D-galactopyranoside (CPRG) as a substrate. Empty bait (pLacZi) and prey (pGADT7) plasmids (−) were used as negative controls. Relative β-galactosidase activity was normalized to the negative control. Data are presented as mean ± SD (n = 3 biological replicates). Statistical analyses were performed using Student's *t*-test and there were no significant differences ($p < 0.05$).

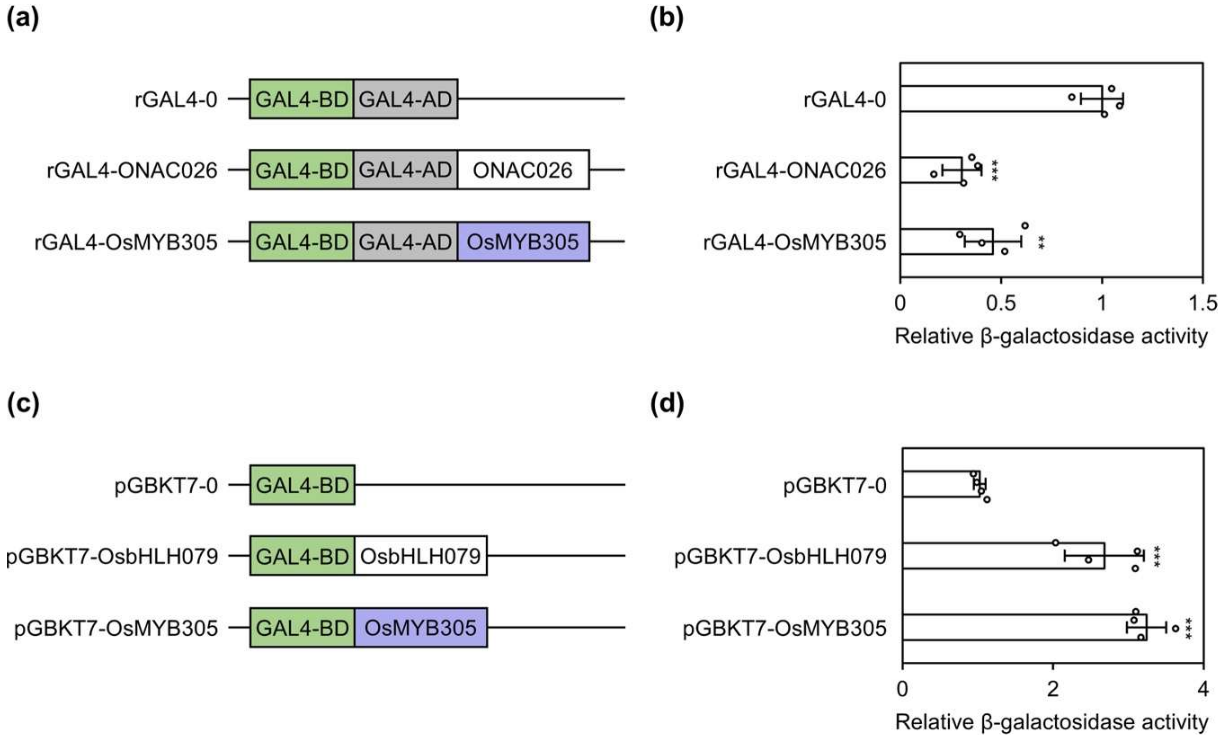

Fig. S10 Transrepression and transactivation activities of OsMYB305. (**a**,**c**) Schematic diagrams of the vectors used for transrepression (a) and transactivation (c) assays. The coding sequences of *ONAC026* and *OsbHLH079* were used as positive controls. BD, binding domain; AD, activation domain. (**b**,**d**) Transcriptional activity was evaluated by measuring β-galactosidase activity using chlorophenol red-β-D-galactopyranoside (CPRG) as a substrate. Empty vectors (rGAL4 or pGBKT7) were used as negative controls. Relative β-galactosidase activity was normalized to the negative control. Data are presented as mean ± SD (n = 4 biological replicates). Statistical analyses were performed using Student's *t*-test and asterisks indicate significant differences (**$p < 0.01$, ***$p < 0.001$).

(a)

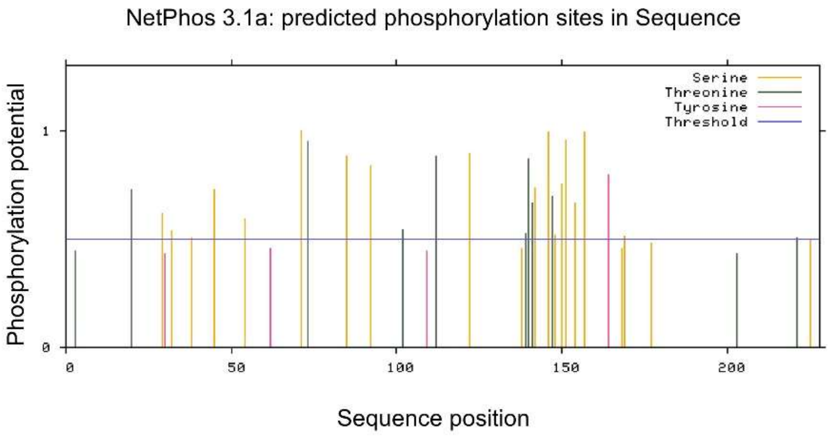

(b)

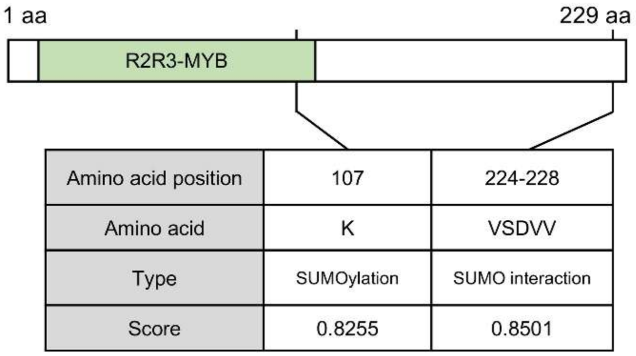

Amino acid position	107	224-228
Amino acid	K	VSDVV
Type	SUMOylation	SUMO interaction
Score	0.8255	0.8501

Fig. S11 Prediction of phosphorylation and SUMOylation site of OsMYB305 *in silico*. Putative phosphorylation and small ubiquitin-like modifier (SUMO) conjugation (SUMOylation) sites in OsMYB305 protein were predicted using NetPhos 3.1 and GPS-SUMO server, respectively. (**a**) Phosphorylation sites were identified at serine, threonine, and tyrosine residues with prediction scores greater than 0.5. (**b**) GPS-SUMO scores indicate the likelihood of SUMOylation or SUMO-interaction site, with higher scores corresponding to greater predicted potential.

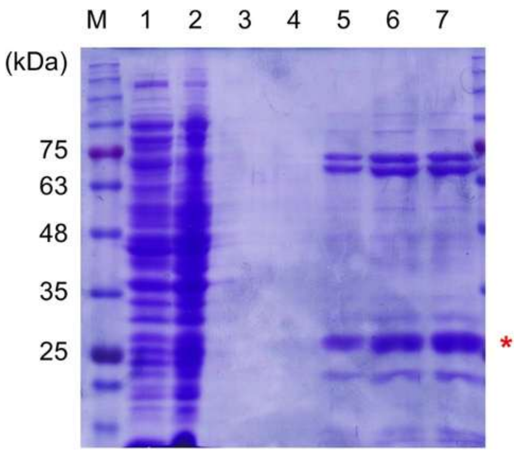

Fig. S12 Purification of recombinant OsMYB305-His protein. Recombinant OsMYB305 fused to 6xHis (containing six consecutive histidine residues) tag was purified, and the SDS-PAGE gel was stained with Coomassie Brilliant Blue. M, molecular marker; lanes 1 and 2, total protein from bacterial cells before (lane 1) and after induction with IPTG (lane 2); lane 3, flow-through fraction from the washing step; lanes 4–7, fractions containing purified OsMYB305–His protein. Asterisk indicates the OsMYB305–His protein.

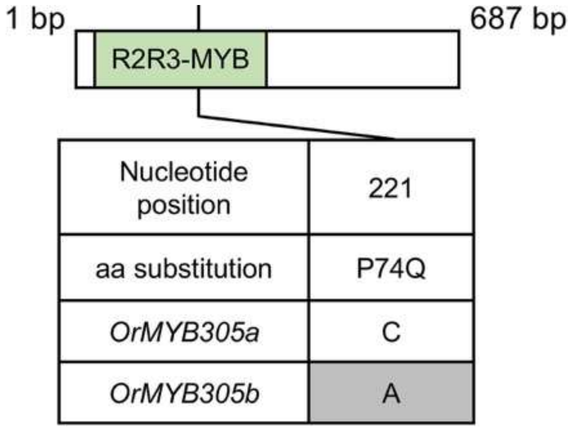

Nucleotide position	221
aa substitution	P74Q
OrMYB305a	C
OrMYB305b	A

Fig. S13 Natural variation of *OrMYB305* in wild rice *Oryza rufipogon*. Analysis of nonsynonymous single nucleotide polymorphisms (SNPs) in *OrMYB305* alleles among 446 *O. rufipogon* accessions. SNP data were obtained from the National Institute of Genetics through the National Bioresource Project. Based on a SNP identified within the R2R3-MYB repeat region of the coding sequence, accessions were classified into two haplotypes: *OrMYB305a* and *OrMYB305b*. The polymorphic nucleotide is indicated in gray.

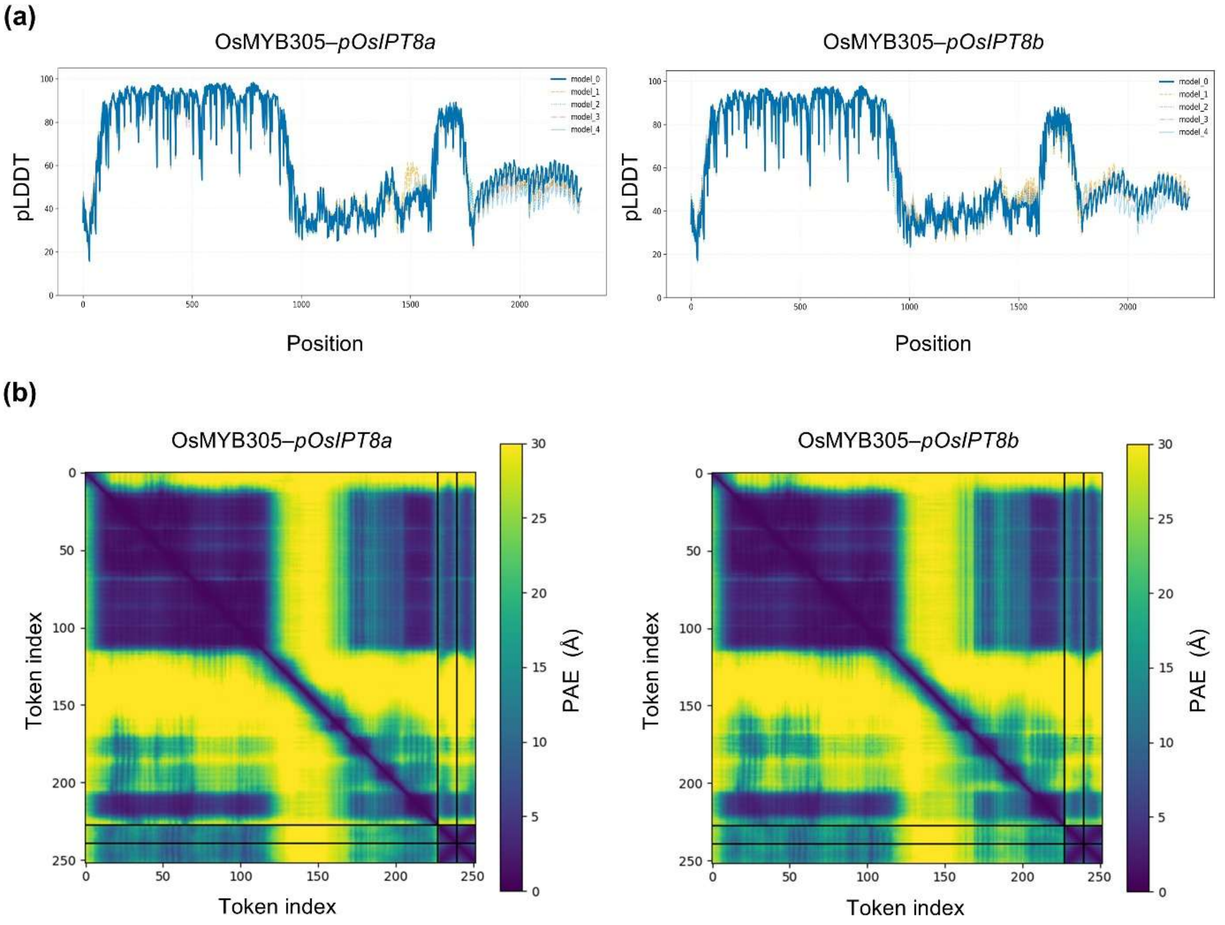

Fig. S14 AlphaFold 3–predicted structures of the OsMYB305–*pOsIPT8* haplotype protein–DNA complexes. (**a**) The structural confidence and positional accuracy were evaluated using the predicted local distance difference Test (pLDDT). Five models were generated for each protein–DNA combination by AlphaFold 3, and the model with the highest pLDDT score was selected for visualization. (**b**) Predicted aligned error (PAE) heatmap of the selected model of the OsMYB305–*pOsIPT8* haplotype complex. Black lines indicate boundaries between the protein chain and the two DNA strands.
